# Supplementary material for: Fair curve designing by Said-Ball curve
Source: PLoS One. 2025 Jul 7;20(7):e0324553. doi: 10.1371/journal.pone.0324553 (PMC12233272; doi:10.1371/journal.pone.0324553)
Supplement: S1 File — (DOCX) [file pone.0324553.s001.docx]

**Appendix**

The formula for Weddle’s rule for $n=6$ is $\int_{0}^{1} f\left( x \right)dx=\frac{3h}{10}\left\{ f\left( 0 \right)+5f\left( \frac{1}{6} \right)+f\left( \frac{2}{6} \right)+ \right.$

$\left. 6f\left( \frac{3}{6} \right)+f\left( \frac{4}{6} \right)+5f\left( \frac{5}{6} \right)+f\left( 1 \right) \right\},$ $h=\frac{1}{6}.$ Integral in Eqn. (4) and (10) are solved by replacing $f\left( x \right)$ by $\left\| P^{'}\left( t \right) \right\|^{2}$ and $\left\| P^{'''}\left( t \right) \right\|^{2}$.

$$\tilde{S}_{1} =\frac{0.0404}{u_{1}}+\frac{8.6708\times{10}^{-4}}{u_{2}}+\frac{2.9297\times{10}^{-3}}{u_{3}}+\frac{8.4675\times{10}^{-7}}{u_{4}}+\frac{4.1345\times{10}^{-9}}{u_{5}}+\frac{1}{20},$$

$$\tilde{S}_{2}=\frac{0.0081}{u_{1}}+\frac{4.3354\times{10}^{-4}}{u_{2}}+\frac{2.9297\times{10}^{-3}}{u_{3}}+\frac{1.6935\times{10}^{-6}}{u_{4}}+\frac{2.0673\times{10}^{-8}}{u_{5}},$$

$$\tilde{S}_{3}=\frac{0.0016}{u_{1}}+\frac{2.1677\times{10}^{-4}}{u_{2}}+\frac{2.9297\times{10}^{-4}}{u_{3}}+\frac{3.3870\times{10}^{-6}}{u_{4}}+\frac{1.0336\times{10}^{-7}}{u_{5}},$$

$$\tilde{S}_{4}=\frac{3.2301\times{10}^{-4}}{u_{1}}+\frac{1.0838\times{10}^{-4}}{u_{2}}+\frac{2.9297\times{10}^{-4}}{u_{3}}+\frac{6.7740\times{10}^{-6}}{u_{4}}+\frac{5.1682\times{10}^{-7}}{u_{5}},$$

$$\tilde{S}_{5}=\frac{6.4602\times{10}^{-5}}{u_{1}}+\frac{5.4192\times{10}^{-5}}{u_{2}}+\frac{2.9297\times{10}^{-4}}{u_{3}}+\frac{1.3548\times{10}^{-5}}{u_{4}}+\frac{2.5841\times{10}^{-6}}{u_{5}},$$

$$\tilde{S}_{6}=\frac{1.2920\times{10}^{-5}}{u_{1}}+\frac{2.7096\times{10}^{-5}}{u_{2}}+\frac{2.9297\times{10}^{-4}}{u_{3}}+\frac{2.7096\times{10}^{-5}}{u_{4}}+\frac{1.2920\times{10}^{-5}}{u_{5}},$$

$$\tilde{S}_{7}=\frac{2.5841\times{10}^{-6}}{u_{1}}+\frac{1.3548\times{10}^{-5}}{u_{2}}+\frac{2.9297\times{10}^{-4}}{u_{3}}+\frac{5.4192\times{10}^{-5}}{u_{4}}+\frac{6.4602\times{10}^{-5}}{u_{5}},$$

$$\tilde{S}_{8}=\frac{5.1682\times{10}^{-7}}{u_{1}}+\frac{6.7740\times{10}^{-6}}{u_{2}}+\frac{2.9297\times{10}^{-4}}{u_{3}}+\frac{1.0838\times{10}^{-4}}{u_{4}}+\frac{3.2301\times{10}^{-4}}{u_{5}},$$

$$\tilde{S}_{9}=\frac{1.0336\times{10}^{-7}}{u_{1}}+\frac{3.3870\times{10}^{-6}}{u_{2}}+\frac{2.9297\times{10}^{-4}}{u_{3}}+\frac{2.1677\times{10}^{-4}}{u_{4}}+\frac{0.0016}{u_{5}},$$

$$\tilde{S}_{10}=\frac{2.0673\times{10}^{-8}}{u_{1}}+\frac{1.6935\times{10}^{-6}}{u_{2}}+\frac{2.9297\times{10}^{-4}}{u_{3}}+\frac{4.3354\times{10}^{-4}}{u_{4}}+\frac{0.0081}{u_{5}},$$

$$\tilde{S}_{11}=\frac{4.1345\times{10}^{-9}}{u_{1}}+\frac{8.4675\times{10}^{-7}}{u_{2}}+\frac{2.9297\times{10}^{-4}}{u_{3}}+\frac{8.6708\times{10}^{-4}}{u_{4}}+\frac{0.0404}{u_{5}}+\frac{1}{20}$$

$u_{1}={({0.2315w}_{1}+0.0463w_{2}+0.7222)}^{4},$ $u_{2}={(0.2963w_{1}+0.1481w_{2}+0.5556)}^{4},$

$u_{3}={(0.25w_{1}+0.25w_{2}+0.5)}^{4},$ $u_{4}={(0.1481w_{1}+0.2963w_{2}+0.5556)}^{4}$

$$u_{5}={(0.0463w_{1}+{0.2315w}_{2}+0.7222)}^{4},$$

$S_{1,1}=8w_{1}^{2},$ $S_{1,2}=0,$ $S_{1,3}=0,$ $S_{1,4}=0,$ $S_{1,5}=0,$ $S_{1,6}=0,$ $S_{2,1}=8w_{1}^{2},$ $S_{2,2}=0,$ $S_{2,3}=0,$

$S_{2,4}=16w_{1}w_{2},$ $S_{2,5}=8w_{1},$ $S_{2,6}=0,$ $S_{3,1}=-16w_{1}^{2}w_{2}-4 w_{1}^{2},$ $S_{3,2}=16w_{2}^{2},$ $S_{3,3}=4,$

$S_{3,4}=16w_{1}^{2}w_{2}+40w_{1}w_{2},$ $S_{3,5}=8w_{1}^{2}+32w_{1}$,$S_{3,6}=16w_{2},$

Similarly, $S_{i,j},i=4,5,\ldots,11, j=1,2,\ldots,6$ are functions of $w_{1}$ and $w_{2}.$ These values can be easily obtained by simplification after numerical integration.

$$M_{1}=\frac{1.2906\times{10}^{-10}}{v_{1}}+\frac{3.3832\times{10}^{-5}}{v_{2}}+\frac{1.1444\times{10}^{-6}}{v_{3}}+\frac{2.4616\times{10}^{-15}}{v_{4}}$$

$$+\frac{0.0094}{v_{5}},$$

$$M_{2}=\frac{2.5812\times{10}^{-10}}{v_{1}}+\frac{1.6916\times{10}^{-5}}{v_{2}}+\frac{1.1444\times{10}^{-6}}{v_{3}}+\frac{1.2308\times{10}^{-14}}{v_{4}}$$

$$+\frac{0.0019}{v_{5}},$$

$$M_{3}=\frac{5.1623\times{10}^{-10}}{v_{1}}+\frac{8.4580\times{10}^{-6}}{v_{2}}+\frac{1.1444\times{10}^{-6}}{v_{3}}+\frac{1.5586\times{10}^{-17}}{v_{4}}$$

$$+\frac{3.7561\times{10}^{-4}}{v_{5}},$$

$$M_{4}=\frac{1.0325\times{10}^{-9}}{v_{1}}+\frac{4,.2290\times{10}^{-6}}{v_{2}}+\frac{1.1444\times{10}^{-6}}{v_{3}}+\frac{3.0770\times{10}^{-13}}{v_{4}}$$

$$+\frac{7.5122\times{10}^{-5}}{v_{5}},$$

$$M_{5}=\frac{2.0649\times{10}^{-9}}{v_{1}}+\frac{2.1145\times{10}^{-6}}{v_{2}}+\frac{1.1444\times{10}^{-6}}{v_{3}}+\frac{1.5385\times{10}^{-12}}{v_{4}}$$

$$+\frac{1.5024\times{10}^{-5}}{v_{5}},$$

$$M_{6}=\frac{4.1299\times{10}^{-9}}{v_{1}}+\frac{1.0572\times{10}^{-6}}{v_{2}}+\frac{1.1444\times{10}^{-6}}{v_{3}}+\frac{7.6925\times{10}^{-12}}{v_{4}}$$

$$+\frac{3.0049\times{10}^{-6}}{v_{5}},$$

$$M_{7}=\frac{8.2598\times{10}^{-9}}{v_{1}}+\frac{5.2862\times{10}^{-7}}{v_{2}}+\frac{1.1444\times{10}^{-6}}{v_{3}}+\frac{3.8463\times{10}^{-11}}{v_{4}}$$

$$+\frac{6.0098\times{10}^{-7}}{v_{5}},$$

$$M_{8}=\frac{1.6520\times{10}^{-8}}{v_{1}}+\frac{2.6431\times{10}^{-7}}{v_{2}}+\frac{1.1444\times{10}^{-6}}{v_{3}}+\frac{1.9231\times{10}^{-10}}{v_{4}}$$

$$+\frac{1.2020\times{10}^{-7}}{v_{5}},$$

$$M_{9}=\frac{3.3039\times{10}^{-8}}{v_{1}}+\frac{1.3216\times{10}^{-7}}{v_{2}}+\frac{1.1444\times{10}^{-6}}{v_{3}}+\frac{9.6156\times{10}^{-10}}{v_{4}}$$

$$+\frac{2.4039\times{10}^{-8}}{v_{5}},$$

$$M_{10}=\frac{6.6078\times{10}^{-8}}{v_{1}}+\frac{6.6078\times{10}^{-8}}{v_{2}}+\frac{1.1444\times{10}^{-6}}{v_{3}}+\frac{4.87078\times{10}^{-9}}{v_{4}}$$

$$+\frac{4.8078\times{10}^{-9}}{v_{5}},$$

$$M_{11}=\frac{1.3216\times{10}^{-7}}{v_{1}}+\frac{3.3039\times{10}^{-8}}{v_{2}}+\frac{1.1444\times{10}^{-6}}{v_{3}}+\frac{2.4039\times{10}^{-8}}{v_{4}}$$

$$+\frac{9.6516\times{10}^{-10}}{v_{5}},$$

$$M_{12}=\frac{2.6431\times{10}^{-7}}{v_{1}}+\frac{1.6520\times{10}^{-8}}{v_{2}}+\frac{1.1444\times{10}^{-6}}{v_{3}}+\frac{1.2020\times{10}^{-7}}{v_{4}}$$

$$+\frac{1.9231\times{10}^{-10}}{v_{5}},$$

$$M_{13}=\frac{5.2862\times{10}^{-7}}{v_{1}}+\frac{8.2598\times{10}^{-9}}{v_{2}}+\frac{1.1444\times{10}^{-6}}{v_{3}}+\frac{6.0098\times{10}^{-7}}{v_{4}}$$

$$+\frac{3.8463\times{10}^{-11}}{v_{5}},$$

$$M_{14}=\frac{1.0572\times{10}^{-6}}{v_{1}}+\frac{4.1299\times{10}^{-9}}{v_{2}}+\frac{1.1444\times{10}^{-6}}{v_{3}}+\frac{3.0049\times{10}^{-6}}{v_{4}}$$

$$+\frac{7.6925\times{10}^{-12}}{v_{5}},$$

$$M_{15}=\frac{2.1145\times{10}^{-6}}{v_{1}}+\frac{2.0649\times{10}^{-9}}{v_{2}}+\frac{1.1444\times{10}^{-6}}{v_{3}}+\frac{1.5024\times{10}^{-5}}{v_{4}}$$

$$+\frac{1.5385\times{10}^{-12}}{v_{5}},$$

$$M_{16}=\frac{4.2290\times{10}^{-6}}{v_{1}}+\frac{1.0325\times{10}^{-9}}{v_{2}}+\frac{1.1444\times{10}^{-6}}{v_{3}}+\frac{7.5122\times{10}^{-5}}{v_{4}}$$

$$+\frac{3.0770\times{10}^{-13}}{v_{5}},$$

$$M_{17}=\frac{8.4580\times{10}^{-6}}{v_{1}}+\frac{5.1623\times{10}^{-10}}{v_{2}}+\frac{1.1444\times{10}^{-6}}{v_{3}}+\frac{3.7561\times{10}^{-4}}{v_{4}}$$

$$+\frac{6.1540\times{10}^{-14}}{v_{5}},$$

$$M_{18}=\frac{1.6916\times{10}^{-5}}{v_{1}}+\frac{2.5812\times{10}^{-10}}{v_{2}}+\frac{1.1444\times{10}^{-6}}{v_{3}}+\frac{0.0019}{v_{4}}$$

$$+\frac{1.2308\times{10}^{-14}}{v_{5}},$$

$$M_{19}=\frac{3.3832\times{10}^{-5}}{v_{1}}+\frac{1.2906\times{10}^{-10}}{v_{2}}+\frac{1.1444\times{10}^{-6}}{v_{3}}+\frac{1.0094}{v_{4}}$$

$$+\frac{2.4616\times{10}^{-15}}{v_{5}}+\frac{1}{20},$$

$$v_{1}={(0.1481w_{1}+0.2963w_{2}+0.5556)}^{8},$$

$$v_{2}={(0.2963w_{1}+0.1481w_{2}+0.5556)}^{8},$$

$$v_{3}={(0.25w_{1}+0.25w_{2}+0.5)}^{8},$$

$$v_{4}={(0.0463w_{1}+0.2315w_{2}+0.7222)}^{8},$$

$$v_{5}={(0.2315w_{1}+0.0463w_{2}+0.7222)}^{8},$$

$$L_{1,1}=1024w_{1}^{6}+1024w_{1}^{5}-1536w_{1}^{4}w_{2}-512w_{1}^{4}-768w_{1}^{3}w_{2}-384w_{1}^{3}+576w_{1}^{2}w_{2}^{2}$$

$$+576w_{1}^{2}w_{2}+144w_{1}^{2},$$

$$L_{1,2}=256w_{1}^{2}w_{2}^{2}-128w_{1}w_{2}^{2}+16w_{2}^{2},$$

$$L_{1,3}=64w_{1}^{2}-128w_{1}+64,$$

$$L_{1,4}=-1024w_{1}^{4}w_{2}-256w_{1}^{3}w_{2}+768w_{1}^{2}w_{2}^{2}+512w_{1}^{2}w_{2}-192w_{1}w_{2}^{2}-96w_{1}w_{2},$$

$$L_{1,5}=-512w_{1}^{4}+256w_{1}^{3}+384w_{1}^{2}w_{2}+448w_{1}^{2}-384w_{1}w_{2}-192w_{1},$$

$$L_{1,6}=256w_{1}^{2}w_{2}-320w_{1}w_{2}+64w_{2}.$$

Similarly, $L_{i,j},i=2,5,\ldots,19, j=1,2,\ldots,6$ are functions of $w_{1}$ and $w_{2}.$

$$S_{4,1}=-32w_{1}^{2}w_{2}-32 w_{1}^{2},$$

$S_{4,2}=32w_{1}w_{2}^{2}+48w_{2}^{2},$ $S_{4,3}=8w_{1}+24,$ $S_{4,4}=16w_{1}w_{2}+32w_{1}^{2}w_{2}-32w_{1}w_{2}^{2},$

$S_{4,5}=32w_{1}^{2}-16w_{1}w_{2}+40w_{1},$ $S_{4,6}=72w_{2}+32w_{1}w_{2},$ $S_{5,1}=16w_{1}^{2}w_{2}^{2}-36 w_{1}^{2},$

$S_{5,2}=16w_{1}^{2}w_{2}^{2}+80w_{1}w_{2}^{2}+52w_{2}^{2},$ $S_{5,3}=4w_{1}^{2}+48w_{1}+60,$

$S_{5,4}=-72w_{1}w_{2}-32w_{1}^{2}w_{2}^{2}-80w_{1}w_{2}^{2},$ $S_{5,5}=-16w_{1}^{2}w_{2}+32w_{1}^{2}-56w_{1}w_{2}-16w_{1},$

$S_{5,6}=16w_{1}^{2}w_{2}+136w_{1}w_{2}+128w_{2},$ $S_{6,1}=32w_{1}^{2}w_{2}^{2}+64 w_{1}^{2}w_{2}+8w_{1}^{2},$

$S_{6,2}=32 w_{1}^{2}w_{2}^{2}+64w_{1}w_{2}^{2}+8w_{2}^{2},$ $S_{6,3}=24w_{1}^{2}+112w_{1}+8w_{2}+80,$

$S_{6,4}=-128w_{1}w_{2}-64w_{1}^{2}w_{2}^{2}-64w_{1}^{2}w_{2}-64w_{1}w_{2}^{2},$

$S_{6,5}=-\left( 64w_{1}^{2}w_{2}+32w_{1}^{2}+80w_{1}w_{2}-104w_{1} \right),$

$S_{6,6}=64w_{1}^{2}w_{2}+208w_{1}w_{2}+16w_{2}^{2}+104 w_{2},$ $S_{7,1}=16w_{1}^{2}w_{2}^{2}+80 w_{1}^{2}w_{2}+52w_{1}^{2},$

$S_{7,2}=16 w_{1}^{2}w_{2}^{2}-36w_{2}^{2},$ $S_{7,3}=52w_{1}^{2}+8w_{1}w_{2}+128w_{1}+32w_{2}+60,$

$$S_{7,4}=-\left( 32w_{1}^{2}w_{2}^{2}+80w_{1}^{2}w_{2}+72w_{1}w_{2} \right),$$

$S_{7,5}=-\left( 80w_{1}^{2}w_{2}+104w_{1}^{2}+16w_{1}w_{2}^{2}+64w_{1}w_{2}+128w_{1} \right),$ $S_{7,6}=0,$

$S_{8,1}=32 w_{1}^{2}w_{2}+48w_{1}^{2},$ $S_{8,2}=-32w_{1}w_{2}^{2}-32w_{2}^{2},$

$S_{8,3}=48w_{1}^{2}+32w_{1}w_{2}+72w_{1}+48w_{2}+24,$

$$S_{8,4}=-32w_{1}^{2}w_{2}^{2}+32w_{1}w_{2}^{2}-48w_{1}w_{2}-72w_{1},$$

$$S_{8,5}=-\left( 80w_{1}^{2}w_{2}+104w_{1}^{2}+16w_{1}w_{2}^{2}+64w_{1}w_{2}+128w_{1} \right),$$

$S_{8,6}=32w_{1}^{2}w_{2}+32w_{1}w_{2}^{2}+32w_{2}^{2}-40 w_{2},$ $S_{9,1}=16 w_{1}^{2},$ $S_{9,2}=-16w_{1}w_{2}^{2}-4w_{2}^{2},$

$S_{9,3}=16w_{1}^{2}+40w_{1}w_{2}+16w_{1}+4w_{2}^{2}+32w_{2}+4,$ $S_{9,4}=16w_{1}w_{2}^{2}+40w_{1}w_{2},$

$S_{9,5}=-32w_{1}^{2}-16w_{1}w_{2}^{2}-40w_{1}-16w_{1},$ $S_{9,6}=16w_{1}w_{2}^{2}-40w_{1}w_{2}-32w_{2},$

$S_{10,1}=16 w_{1}^{2},$ $S_{10,2}=8w_{2}^{2},$ $S_{10,3}=8w_{2}^{2}+8w_{2}+16w_{1}w_{2},$ $S_{10,4}=16w_{1}w_{2},$

$S_{10,5}=-16w_{1}w_{2},$ $S_{10,6}=-\left( 16w_{2}^{2}+16w_{1}w_{2}+8w_{2} \right),$ $S_{11,1}=0,$ $S_{11,2}=4w_{2}^{2},$

$S_{11,3}=4w_{2}^{2},$ $S_{11,4}=0,$ $S_{11,5}=0,$ $S_{11,6}=-8w_{2}^{2}.$

$$L_{2,1}=4096w_{1}^{6}+7168w_{1}^{5}w_{2}+7680w_{1}^{5}-4096w_{1}^{4}w_{2}-4096w_{1}^{4}-5376w_{1}^{3}w_{2}^{2}$$

$$-9216w_{1}^{3}w_{2}-4800w_{1}^{3}+3456w_{1}^{2}w_{2}^{2}+5760w_{1}^{2}w_{2}+2016w_{1}^{2},$$

$$L_{2,2}=256w_{1}^{2}w_{2}^{2}+2560w_{1}w_{2}^{3}+576w_{1}w_{2}^{2}-640w_{2}^{3}-160w_{2}^{2},$$

$$L_{2,3}=-128w_{1}^{2}+640w_{1}w_{2}-384w_{1}-640w_{2}+512,$$

$$L_{2,4}=-2560w_{1}^{4}w_{2}-8704w_{1}^{3}w_{2}^{2}-3840w_{1}^{3}w_{2}+1024w_{1}^{2}w_{2}^{2}+2944w_{1}^{2}w_{2}$$

$$+3840w_{1}w_{2}^{3}+2304w_{1}w_{2}^{2}-192w_{1}w_{2},$$

$$L_{2,5}=-512w_{1}^{4}-4352w_{1}^{3}w_{2}+1920w_{1}w_{2}^{2} -1728w_{1}w_{2}-2112w_{1},$$

$$L_{2,6}=-128w_{1}^{2}w_{2}+2560w_{1}w_{2}^{2}-768w_{1}w_{2}-1600w_{2}^{2}-64w_{2},$$

$$L_{3,1}=6144w_{1}^{6}+25600w_{1}^{5}w_{2}+20480w_{1}^{5}+24832w_{1}^{4}w_{2}^{2}+20736w_{1}^{4}w_{2}$$

$$-11968w_{1}^{4}-18432w_{1}^{4}w_{2}^{2}-38784w_{1}^{3}w_{2}-24000w_{1}^{3}-9216w_{1}^{2}w_{2}^{3}$$

$$-4032w_{1}^{2}w_{2}^{2}+19008w_{1}^{2}w_{2}+11664w_{1}^{2},$$

$$L_{3,2}=-1536w_{1}^{3}w_{2}^{2} +1536w_{1}^{2}w_{2}^{3}-1344w_{1}^{2}w_{2}^{2}+9856w_{1}w_{2}^{3}-640w_{2}+7424w_{1}w_{2}^{2}$$

$$+6400w_{2}^{4}+960w_{2}^{3}-1264w_{2}^{2},$$

$$L_{3,3}=-768w_{1}^{3}+384w_{1}^{2}w_{2}-1920w_{1}^{2}+1984w_{1}w_{2}+3392w_{1}+1600w_{2}^{2}-5568w_{2}$$

$$+896,$$

$$L_{3,4}=3072w_{1}^{5}w_{2}-3072w_{1}^{4}w_{2}^{2}+3072w_{1}^{4}w_{2}-39424w_{1}^{3}w_{2}^{2}-20480w_{1}^{3}w_{2}$$

$$-21760w_{1}^{2}w_{2}^{3}-29568w_{1}^{2}w_{2}^{2}-3008w_{1}^{2}w_{2}+26496w_{1}w_{2}^{3}+33792w_{1}w_{2}^{2}$$

$$+6816w_{1}w_{2},$$

$$L_{3,5}=3072w_{1}^{5}-1536w_{1}^{4}w_{2}+13056w_{1}^{4}-21248w_{1}^{3}w_{2}+7808w_{1}^{3}-10880w_{1}^{2}w_{2}^{2}$$

$$-13440w_{1}^{2}w_{2}+5024w_{1}^{2}+17856w_{1}w_{2}^{2}+8544w_{1}w_{2}-8256w_{1},$$

$$L_{3,6}=-2304w_{1}^{3}w_{2}+1536w_{1}^{2}w_{2}^{2}-5376w_{1}^{2}w_{2}+8896w_{1}w_{2}^{2}+5024w_{1}w_{2}$$

$$+6400w_{2}^{3}-9504w_{2}^{2}-4672w_{2},$$

$$L_{4,1}=2048w_{1}^{6}-2048w_{1}^{6}w_{2}+2048w_{1}^{5}w_{2}^{2}+29696w_{1}^{5}w_{2}+13312w_{1}^{5}$$

$$+98304w_{1}^{4}w_{2}^{2}+116736w_{1}^{4}w_{2}-15616w_{1}^{4}+41472w_{1}^{3}w_{2}^{3}+44288w_{1}^{3}w_{2}^{2}$$

$$-45440w_{1}^{3}w_{2}-54976w_{1}^{3}-51456w_{1}^{2}w_{2}^{3}-92928w_{1}^{2}w_{2}^{2}-2112w_{1}^{2}w_{2}$$

$$+34176w_{1}^{2},$$

$$L_{4,2}=-1536w_{1}^{3}w_{2}^{2}+1536w_{1}^{2}w_{2}^{3}-1344w_{1}^{2}w_{2}^{2}+9856w_{1}w_{2}^{3}-640w_{2}+7424w_{1}w_{2}^{2}$$

$$+6400w_{2}^{4}+960w_{2}^{3}-1264w_{2}^{2},$$

$$L_{4,3}=-512w_{1}^{4}+256w_{1}^{3}w_{2}-3456w_{1}^{3}-1664{w_{1}^{2}w}_{2}+5248w_{1}^{2}+1152w_{1}w_{2}^{2}$$

$$-16832w_{1}w_{2}+20160w_{1}+15808w_{2}^{2}-15680w_{2}-4480,$$

$$L_{4,4}=15360w_{1}^{5}w_{2}-12288w_{1}^{4}w_{2}^{2}+11264w_{1}^{4}w_{2}-4096w_{1}^{2}w_{2}^{3}-78848w_{1}^{3}w_{2}^{2}$$

$$-68608w_{1}^{3}w_{2}-88066w_{1}^{2}w_{2}^{3}-145408w_{1}^{2}w_{2}^{2}-61824w_{1}^{2}w_{2}-35328w_{1}w_{2}^{4}$$

$$+32768w_{1}w_{2}^{3}+144256w_{1}w_{2}^{2}+55808w_{1}w_{2}+2048w_{1}^{6}w_{2}-2048w_{1}^{5}w_{2}^{2},$$

$$L_{4,5}=2048w_{1}^{6}-1024w_{1}^{5}w_{2}+26112w_{1}^{5}-5120w_{1}^{4}w_{2}+61440w_{1}^{4}-2048w_{1}^{3}w_{2}^{2}$$

$$-33792w_{1}^{3}w_{2}+14336w_{1}^{3}-47104w_{1}^{2}w_{2}^{2}-84736w_{1}^{2}w_{2}-41728w_{1}^{2}$$

$$-17664w_{1}w_{2}^{3}+51328w_{1}w_{2}^{2}+84224w_{1}w_{2}-6272w_{1},$$

$$L_{4,6}=-1536w_{1}^{4}w_{2}+1024w_{1}^{3}w_{2}^{2}-10752w_{1}^{3}w_{2}+768w_{2}^{2}-4864w_{1}^{2}w_{2}$$

$$+4608w_{1}w_{2}^{3}-27648w_{1}w_{2}^{2}+11264w_{1}w_{2}+54400w_{2}^{3}-3072w_{1}^{2}w_{2}^{2}$$

$$-24192w_{2},$$

$$L_{5,1}=3072w_{1}^{5}w_{2}^{2}-13312w_{1}^{6}-8192w_{1}^{6}w_{2}+4608w_{1}^{5}w_{2}-42496w_{1}^{5}+5120w_{1}^{4}w_{2}^{3}$$

$$+151296w_{1}^{4}w_{2}^{2}+219392w_{1}^{4}w_{2}-6272w_{1}^{4}+168448w_{1}^{3}w_{2}^{3}+331008w_{1}^{3}w_{2}^{2}$$

$$+136320w_{1}^{3}w_{2}-21824w_{1}^{3}+38400w_{1}^{2}w_{2}^{4}-52224w_{1}^{2}w_{2}^{3}-319488w_{1}^{2}w_{2}^{2}$$

$$-194496w_{1}^{2}w_{2}+41280w_{1}^{2},$$

$$L_{5,2}=-256w_{1}^{4}w_{2}^{2}-6144w_{1}^{3}w_{2}^{3}+2560w_{1}^{3}w_{2}^{2}+6400w_{1}^{2}w_{2}^{4}-37632w_{1}^{2}w_{2}^{3}$$

$$+5400w_{1}^{2}w_{2}^{2}+56832w_{1}w_{2}^{4}+16768w_{1}w_{2}^{3}+8000w_{1}w_{2}^{2}-15360w_{2}^{5}$$

$$+122432w_{2}^{4}+221120w_{2}^{3}+60480w_{2}^{2},$$

$$L_{5,3}=256w_{1}^{4}-4096w_{1}^{3}w_{2}+17408w_{1}^{3}+1600w_{1}^{2}w_{2}^{2}-51776w_{1}^{2}w_{2}+61968w_{1}^{2}$$

$$+22208w_{1}w_{2}^{2}-109664w_{1}w_{2}+22912w_{1}-3840w_{2}^{3}+61776w_{2}^{2}+5888w_{2}$$

$$-24640,$$

$$L_{5,4}=8192w_{1}^{6}w_{2}+27136w_{1}^{5}w_{2}-5120w_{1}^{4}w_{2}^{3}-23552w_{1}^{4}w_{2}^{2}-29952w_{1}^{4}w_{2}$$

$$-13824w_{1}^{3}w_{2}^{3}-3072w_{1}^{5}w_{2}^{2}-120320w_{1}^{3}w_{2}^{2}-192896w_{1}^{3}w_{2}-2560w_{1}^{2}w_{2}^{4}$$

$$-100096w_{1}^{2}w_{2}^{3}-217472w_{1}^{2}w_{2}^{2}-167936w_{1}^{2}w_{2}-211712w_{1}w_{2}^{4}$$

$$-252672w_{1}w_{2}^{3}+224256w_{1}w_{2}^{2}+200320w_{1}w_{2},$$

$$L_{5,5}=14336w_{1}^{6}+1024w_{1}^{5}w_{2}+85504w_{1}^{5}-2560w_{1}^{4}w_{2}^{2}+30208w_{1}^{4}w_{2}+125824w_{1}^{4}$$

$$-8192w_{1}^{3}w_{2}^{2}-10240w_{1}^{3}w_{2}-47232w_{1}^{3}-1280w_{1}^{2}w_{2}^{3}-14208w_{1}^{2}w_{2}^{2}$$

$$-118720w_{1}^{2}w_{2}-205632w_{1}^{2}-125056w_{1}w_{2}^{3}-32448w_{1}w_{2}^{2}+249792w_{1}w_{2}$$

$$+58880w_{1},$$

$$L_{5,6}=-1536w_{1}^{4}w_{2}-11264w_{1}^{3}w_{2}^{2}+5376w_{1}^{3}w_{2}+6400w_{1}^{2}w_{2}^{3}-114688w_{1}^{2}w_{2}^{2}$$

$$+72832w_{1}w_{2}^{3}-167360w_{1}w_{2}^{2}-82752w_{1}w_{2}-15360w_{2}^{4}+179968w_{2}^{3}$$

$$+162752w_{2}^{2}+5312w_{1}^{2}w_{2}-39680w_{2},$$

$$L_{6,1}=12288w_{1}^{4}w_{2}^{3}-36864w_{1}^{6}-5120w_{1}^{5}w_{2}^{2}-32768w_{1}^{5}w_{2}-108032w_{1}^{5}$$

$$-12288w_{1}^{6}w_{2}+32768w_{1}^{5}w_{2}-108032w_{1}^{5}-12288w_{1}^{6}w_{2}+128512w_{1}^{4}w_{2}^{2}$$

$$+205824w_{1}^{4}w_{2}+5120w_{1}^{3}+32000w_{1}^{4}+38400w_{1}^{3}w_{2}^{4}+246784w_{1}^{3}w_{2}^{3}$$

$$+615936w_{1}^{3}w_{2}^{2}+182464w_{1}^{3}+198144w_{1}^{2}w_{2}^{4}+326400w_{1}^{2}w_{2}^{3}$$

$$-356736w_{1}^{2}w_{2}^{2}-604608w_{1}^{2}w_{2}+486656w_{1}^{3}w_{2}-49920w_{1}^{2},$$

$$L_{6,2}=3072w_{1}^{5}w_{2}^{2}-6144w_{1}^{4}w_{2}^{3}+13824w_{1}^{4}w_{2}^{2}+3072w_{1}^{3}w_{2}^{4}-62464w_{1}^{3}w_{2}^{3}$$

$$-1280w_{1}^{3}w_{2}^{2}+62976w_{1}^{2}w_{2}^{4}-89088w_{1}^{2}w_{2}^{3}-74880w_{1}^{2}w_{2}^{2}-14336w_{1}w_{2}^{5}$$

$$+217088w_{1}w_{2}^{4}+319744w_{1}w_{2}^{3}+34496w_{1}w_{2}^{2}-89600w_{2}^{5}+94464w_{2}^{4}$$

$$+557120w_{2}^{3}+265728w_{2}^{2},$$

$$L_{6,3}=3072w_{1}^{5}-3072w_{1}^{4}w_{2}+38400w_{1}^{4}+768w_{1}^{3}w_{2}^{2}-63488w_{1}^{3}w_{2}+151616w_{1}^{3}$$

$$+23936w_{1}^{2}w_{2}^{2}-265088w_{1}^{2}w_{2}+134304w_{1}^{2}-3584w_{1}w_{2}^{3}+112064w_{1}w_{2}^{2}$$

$$-214272w_{1}w_{2}-103872w_{1}-27648w_{2}^{3}+109024w_{2}^{2}+148032w_{2}-40192,$$

$$L_{6,4}=12288w_{1}^{6}w_{2}+2048w_{1}^{5}w_{2}^{2}+1024w_{1}^{5}w_{2}-6144w_{1}^{4}w_{2}^{3}-49152w_{1}^{4}w_{2}^{2}$$

$$+27648w_{1}^{3}w_{2}^{3}-120832w_{1}^{3}w_{2}^{2}-397824w_{1}^{3}w_{2}-94208w_{1}^{2}w_{2}^{4}$$

$$+57344w_{1}^{2}w_{2}^{2}-50560w_{1}^{2}w_{2}+41984w_{1}w_{2}^{5}-470528w_{1}w_{2}^{4}-1211904w_{1}w_{2}^{3}$$

$-195072w_{1}^{4}w_{2}-266368w_{1}w_{2}^{2}+376576w_{1}w_{2}-8192w_{1}^{3}w_{2}^{4}-115712w_{1}^{2}w_{2}^{3}$,

$$L_{6,5}=36864w_{1}^{6}+38912w_{1}^{5}w_{2}+142336w_{1}^{5}+1024w_{1}^{4}w_{2}^{2}+151040w_{1}^{4}w_{2}$$

$$+118272w_{1}^{3}w_{2}^{2}+128000w_{1}^{3}w_{2}-339328w_{1}^{3}-70656w_{1}^{2}w_{2}^{3}+189184w_{1}^{2}w_{2}^{2}$$

$$+278400w_{1}^{2}w_{2}-341632w_{1}^{2}+20992w_{1}w_{2}^{4}-338176w_{1}w_{2}^{3}-560256w_{1}w_{2}^{2}$$

$$+82176w_{1}^{4}-4096w_{1}^{3}w_{2}^{3}+228736w_{1}w_{2}+238208w_{1},$$

$$L_{6,6}=6144w_{1}^{5}w_{2}-9216w_{1}^{4}w_{2}^{2}+46080w_{1}^{4}w_{2}+3072w_{1}^{3}w_{2}^{3}-148992w_{1}^{3}w_{2}^{2}$$

$$+72960w_{1}^{3}w_{2}+79360w_{1}^{2}w_{2}^{3}-484864w_{1}^{2}w_{2}^{2}-206592w_{1}^{2}w_{2}-14336w_{1}w_{2}^{4}$$

$$+321536w_{1}w_{2}^{3}-99712w_{1}w_{2}^{2}-415616w_{1}w_{2} -100096w_{2}^{4}+238592w_{2}^{3}$$

$$+634240w_{2}^{2}+77440w_{2},$$

$$L_{7,1}=1024w_{1}^{6}w_{2}^{2}-6144w_{1}^{6}w_{2}-44032w_{1}^{6}-2048w_{1}^{5}w_{2}^{3}-12288w_{1}^{5}w_{2}^{2}$$

$$-97280w_{1}^{5}+1024w_{1}^{4}w_{2}^{4}-12288w_{1}^{4}w_{2}^{3}+13056w_{1}^{4}w_{2}^{2}+106496w_{1}^{4}w_{2}$$

$$+43008w_{1}^{3}w_{2}^{4}+217088w_{1}^{3}w_{2}^{3}+350464w_{1}^{3}w_{2}^{2}+399488w_{1}^{3}w_{2}+422720w_{1}^{3}$$

$$-12288w_{1}^{2}w_{2}^{5}+398080w_{1}^{2}w_{2}^{4}+1286656w_{1}^{2}w_{2}^{3}+560832w_{1}^{2}w_{2}^{2}$$

$$-57344w_{1}^{5}w_{2}-773696w_{1}^{2}w_{2}+164800w_{1}^{4}-274112w_{1}^{2},$$

$$L_{7,2}=1024w_{1}^{6}w_{2}^{2}-2048w_{1}^{5}w_{2}^{3}+14336w_{1}^{5}w_{2}^{2}+1024w_{1}^{4}w_{2}^{4}-38912w_{1}^{4}w_{2}^{3}$$

$$+14848w_{1}^{4}w_{2}^{2}+33792w_{1}^{3}w_{2}^{4}-153088w_{1}^{3}w_{2}^{3}-151552w_{1}^{3}w_{2}^{2}-9216w_{1}^{2}w_{2}^{5}$$

$$+311552w_{1}w_{2}^{4}+1202560w_{1}^{2}w_{2}^{3}+225024w_{1}^{2}w_{2}^{4}+181504w_{1}^{2}w_{2}^{3}$$

$$-235776w_{1}^{2}w_{2}^{2}-95744w_{1}w_{2}^{5}+441024w_{1}w_{2}^{2}+9216w_{2}^{6}-206336w_{2}^{5}$$

$$-255616w_{2}^{4}+735296w_{2}^{3}+650304w_{2}^{2},$$

$$L_{7,3}=1024w_{1}^{6}-1024w_{1}^{5}w_{2}+34304w_{1}^{5}+256w_{1}^{4}w_{2}^{2}-34560w_{1}^{4}w_{2}+216640w_{1}^{4}$$

$$+13312w_{1}^{3}w_{2}^{2}-278912w_{1}^{3}w_{2}+380608w_{1}^{3}-2304w_{1}^{2}w_{2}^{3}+111616w_{1}^{2}w_{2}^{2}$$

$$-542976w_{1}^{2}w_{2}-94096w_{1}^{2}-31232w_{1}w_{2}^{3}+225152w_{1}w_{2}^{2}+32992w_{1}w_{2}$$

$$+2304w_{2}^{4}-82368w_{2}^{3}+25776w_{2}^{2}+410752w_{2}-418304w_{1}+31040,$$

$$L_{7,4}=-2048w_{1}^{6}w_{2}^{2}+6144w_{1}^{6}w_{2}+4096w_{1}^{5}w_{2}^{3}-2048w_{1}^{5}w_{2}^{2}-90112w_{1}^{5}w_{2}$$

$$-2048w_{1}^{4}w_{2}^{4}+51200w_{1}^{4}w_{2}^{3}-55808w_{1}^{4}w_{2}^{2}-440064w_{1}^{4}w_{2}-76800w_{1}^{3}w_{2}^{4}$$

$$+68096w_{1}^{3}w_{2}^{3}+149760w_{1}^{3}w_{2}^{2}-342784w_{1}^{3}w_{2}+21504w_{1}^{2}w_{2}^{5}$$

$$-379904w_{1}^{2}w_{2}^{4}-927744w_{1}^{2}w_{2}^{3}+129920w_{1}^{2}w_{2}^{2}+610816w_{1}^{2}w_{2}$$

$$+209408w_{1}w_{2}^{5}-280832w_{1}w_{2}^{4}-2396800w_{1}w_{2}^{3}-1852416w_{1}w_{2}^{2}$$

$$+252032w_{1}w_{2}$$

$$L_{7,5}=-2048w_{1}^{6}w_{2}+43008w_{1}^{6}+3072w_{1}^{5}w_{2}^{2}+103936w_{1}^{5}w_{2}+90624w_{1}^{5}$$

$$-1024w_{1}^{4}w_{2}^{3}+140288w_{1}^{4}w_{2}^{2}+323328w_{1}^{4}w_{2}-270464w_{1}^{4}-49664w_{1}^{3}w_{2}^{3}$$

$$+593920w_{1}^{3}w_{2}^{2}+744320w_{1}^{3}w_{2}-746368w_{1}^{3}+10752w_{1}^{2}w_{2}^{4}-270848w_{1}^{2}w_{2}^{3}$$

$$+86016w_{1}^{2}w_{2}^{2}+1071040w_{1}^{2}w_{2}+46144w_{1}^{2}+110848w_{1}w_{2}^{4}-345984w_{1}w_{2}^{3}$$

$$-1513024w_{1}w_{2}^{2}-568000w_{1}w_{2}+400128w_{1},$$

$$L_{7,6}=1024w_{1}^{4}w_{2}^{3}-83456w_{1}^{4}w_{2}^{2}+173312w_{1}^{4}w_{2} +43520w_{1}^{3}w_{2}^{3}-543744w_{1}^{3}w_{2}^{2}$$

$$-131200w_{1}^{3}w_{2}-9216w_{1}^{2}w_{2}^{4}+325632w_{1}^{2}w_{2}^{3}-703488w_{1}^{2}w_{2}^{2}$$

$$-1120704w_{1}^{2}w_{2}-110336w_{1}w_{2}^{4}+563328w_{1}w_{2}^{3}+917952w_{1}w_{2}^{2}$$

$$-664000w_{1}w_{2}+9216w_{2}^{5}-266368w_{2}^{4}-133888w_{2}^{3}+1160512w_{2}^{2}$$

$$+524288w_{2},$$

$$L_{8,1}=4096w_{1}^{6}w_{2}^{2}+12288w_{1}^{6}w_{2}-16384w_{1}^{6}-10240w_{1}^{5}w_{2}^{3}-25600w_{1}^{5}w_{2}^{2}$$

$$-24576w_{1}^{5}w_{2}+35840w_{1}^{5}+8192w_{1}^{4}w_{2}^{4}-56320w_{1}^{4}w_{2}^{3}-307200w_{1}^{4}w_{2}^{2}$$

$$-160256w_{1}^{4}w_{2}+358400w_{1}^{4}-2048w_{1}^{3}w_{2}^{5}+125952w_{1}^{3}w_{2}^{4}+423936w_{1}^{3}w_{2}^{3}$$

$$-63488w_{1}^{3}w_{2}^{2}-490112w_{1}^{3}w_{2}+256576w_{1}^{3}-56320w_{1}^{2}w_{2}^{5}+326656w_{1}^{2}w_{2}^{4}$$

$$+2109440w_{1}^{2}w_{2}^{3}-56320w_{1}^{2}w_{2}^{5}+326656w_{1}^{2}w_{2}^{4}+2109440w_{1}^{2}w_{2}^{3}$$

$$+2497280w_{1}^{2}w_{2}^{2}+78016w_{1}^{2}w_{2}-441472w_{1}^{2},$$

$$L_{8,2}=4096w_{1}^{6}w_{2}^{2}-10240w_{1}^{5}w_{2}^{3}+19456w_{1}^{5}w_{2}^{2}+8192w_{1}^{4}w_{2}^{4}-89088w_{1}^{4}w_{2}^{3}$$

$$-100352w_{1}^{4}w_{2}^{2}-2048w_{1}^{3}w_{2}^{5}+117760w_{1}^{3}w_{2}^{4}-40448w_{1}^{3}w_{2}^{3}-480512w_{1}^{3}w_{2}^{2}$$

$$-54272w_{1}^{2}w_{2}^{5}+384160w_{1}^{2}w_{2}^{4}+1136896w_{1}^{2}w_{2}^{3}+38656w_{1}^{2}w_{2}^{2}+6144w_{1}w_{2}^{6}$$

$$-246784w_{1}w_{2}^{5}-100352w_{1}w_{2}^{4} +2070912w_{1}w_{2}^{3}+1576768w_{1}w_{2}^{2}$$

$$+43008w_{2}^{6}-200704w_{2}^{5}-769280w_{2}^{4}+313280w_{2}^{3}+993408w_{2}^{2},$$

$$L_{8,3}=10240w_{1}^{6}-9216w_{1}^{5}w_{2}+150016w_{1}^{5}+3072w_{1}^{4}w_{2}^{2}-128000w_{1}^{4}w_{2}$$

$$+518656w_{1}^{4}+3072w_{1}^{4}w_{2}^{2}-12800w_{1}^{4}w_{2}+518656w_{1}^{4}-512w_{1}^{3}w_{2}^{3}$$

$$+61184w_{1}^{3}w_{2}^{2}-484992w_{1}^{3}w_{2}+236352w_{1}^{3}-17152w_{1}^{2}w_{2}^{3}$$

$$+202240w_{1}^{2}w_{2}^{2}-250688w_{1}^{2}w_{2}-937408w_{1}^{2}+1536w_{1}w_{2}^{4}-98944w_{1}w_{2}^{3}$$

$$+96704w_{1}w_{2}^{2}+825536w_{1}w_{2}-595264w_{1}+11520w_{2}^{4}-130880w_{2}^{3}$$

$$-258176w_{2}^{2}+527552w_{2}+266624,$$

$$L_{8,4}=8192w_{1}^{6}w_{2}^{2}-12288w_{1}^{6}w_{2}+20480w_{1}^{5}w_{2}^{3}+6144w_{1}^{5}w_{2}^{2}-194560w_{1}^{5}w_{2}$$

$$-16384w_{1}^{4}w_{2}^{4}+145408w_{1}^{4}w_{2}^{3}+167936w_{1}^{4}w_{2}^{2}-408064w_{1}^{4}w_{2}$$

$$+4096w_{1}^{3}w_{2}^{5}-243712w_{1}^{3}w_{2}^{4}-442368w_{1}^{3}w_{2}^{3}+454656w_{1}^{3}w_{2}^{2}$$

$$+550912w_{1}^{3}w_{2}+110592w_{1}^{2}w_{2}^{5}-516096w_{1}^{2}w_{2}^{4}-2921472w_{1}^{2}w_{2}^{3}$$

$$-1714176w_{1}^{2}w_{2}^{2}+1263232w_{1}^{2}w_{2}-6144w_{1}w_{2}^{6}+416768w_{1}w_{2}^{5}$$

$$+714752w_{1}w_{2}^{4}-2251776w_{1}w_{2}^{3}-3770496w_{1}w_{2}^{2}-514560w_{1}w_{2},$$

$$L_{8,5}=-14336w_{1}^{6}w_{2}+6144w_{1}^{6}+22528w_{1}^{5}w_{2}^{2}+92160w_{1}^{5}w_{2}-172032w_{1}^{5}$$

$$-10240w_{1}^{4}w_{2}^{3}+512000w_{1}^{4}w_{2}^{2}+673280w_{1}^{4}w_{2}-821248w_{1}^{4}+2048w_{1}^{3}w_{2}^{4}$$

$$-98304w_{1}^{3}w_{2}^{3}+990208w_{1}^{3}w_{2}^{2}+1935616w_{1}^{3}w_{2}-426496w_{1}^{3}+59392w_{1}^{2}w_{2}^{4}$$

$$-271360w_{1}^{2}w_{2}^{3}-948736w_{1}^{2}w_{2}^{2}+794112w_{1}^{2}w_{2}+1099264w_{1}^{2}-3072w_{1}w_{2}^{5}$$

$$+253440w_{1}w_{2}^{4}+247808w_{1}w_{2}^{3}-1933440w_{1}w_{2}^{2}-2172928w_{1}w_{2}$$

$$+184192w_{1},$$

$$L_{8,6}=14336w_{1}^{6}w_{2}-22528w_{1}^{5}w_{2}^{2}+104960w_{1}^{4}w_{2}-2048w_{1}^{3}w_{2}^{4}+177152w_{1}^{3}w_{2}^{3}$$

$$-820736w_{1}^{3}w_{2}^{2}-1287936w_{1}^{3}w_{2}-61440w_{1}^{2}w_{2}^{4}+567808w_{1}^{2}w_{2}^{3}$$

$$+299520w_{1}^{2}w_{2}^{2}-2386432w_{1}^{2}w_{2}+6144w_{1}w_{2}^{5}-319488w_{1}w_{2}^{4}+89856w_{1}w_{2}^{3}$$

$$+2606336w_{1}w_{2}^{2}+193792w_{1}w_{2}+44544w_{2}^{5}-346624w_{2}^{4}-952192w_{2}^{3}$$

$$+963072w_{2}^{2}+1250688w_{2},$$

$$L_{9,1}=6144w_{1}^{6}w_{2}^{2}+36864w_{1}^{6}w_{2}+33792w_{1}^{6}-20480w_{1}^{5}w_{2}^{3}-83968w_{1}^{5}w_{2}^{2}$$

$$-7168w_{1}^{5}w_{2}+219136w_{1}^{5}+23552w_{1}^{4}w_{2}^{4}-14336w_{1}^{4}w_{2}^{3}-628224w_{1}^{4}w_{2}^{2}$$

$$-791552w_{1}^{4}w_{2}-10240w_{1}^{3}w_{2}^{5}+161792w_{1}^{3}w_{2}^{4}+902144w_{1}^{3}w_{2}^{3}$$

$$+563200w_{1}^{3}w_{2}^{2}-1061248w_{1}^{3}w_{2}-419136w_{1}^{3}+1024w_{1}^{2}w_{2}^{6}-100352w_{1}^{2}w_{2}^{5}$$

$$-77824w_{1}^{2}w_{2}^{4}+1737216w_{1}^{2}w_{2}^{3}+3914752w_{1}^{2}w_{2}^{2}+1875392w_{1}^{2}w_{2}$$

$$-231712w_{1}^{2}+272384w_{1}^{4},$$

$$L_{9,2}=6144w_{1}^{6}w_{2}^{2}-20480w_{1}^{5}w_{2}^{3}-16384w_{1}^{5}w_{2}^{2}+23552w_{1}^{4}w_{2}^{4}-67584w_{1}^{4}w_{2}^{3}$$

$$-332288w_{1}^{4}w_{2}^{2}-10240w_{1}^{3}w_{2}^{5}+184320w_{1}^{3}w_{2}^{4}+477184w_{1}^{3}w_{2}^{3}$$

$$+1024w_{1}^{2}w_{2}^{6}-126976w_{1}^{2}w_{2}^{5}+92928w_{1}^{2}w_{2}^{4}+2134528w_{1}^{2}w_{2}^{3}$$

$$+26624w_{1}w_{2}^{6}-295936w_{1}w_{2}^{5}-937728w_{1}w_{2}^{4}+1629568w_{1}w_{2}^{3}$$

$$+80896w_{2}^{6}+22528w_{2}^{5}-848704w_{2}^{4}-521536w_{2}^{3}+922848w_{2}^{2}$$

$$+2942272w_{1}w_{2}^{2}+1419968w_{1}^{2}w_{2}^{2}-485376w_{1}^{3}w_{2}^{2},$$

$$L_{9,3}=39936w_{1}^{6}-23552w_{1}^{5}w_{2}+326144w_{1}^{5}+9216w_{1}^{4}w_{2}^{2}-143872w_{1}^{4}w_{2}$$

$$+524800w_{1}^{4}-3072w_{1}^{3}w_{2}^{3}+100096w_{1}^{3}w_{2}^{2}-77824w_{1}^{3}w_{2}-785472w_{1}^{3}$$

$$+256w_{1}^{2}w_{2}^{4}-37376w_{1}^{2}w_{2}^{3}+103808w_{1}^{2}w_{2}^{2}+884480w_{1}^{2}w_{2}-1838256w_{1}^{2}$$

$$+6912w_{1}w_{2}^{4}-167808w_{1}w_{2}^{3}-361984w_{1}w_{2}^{2}+1361952w_{1}w_{2}-79232w_{1}$$

$$+25664w_{2}^{4}-118080w_{2}^{3}-526576w_{2}^{2}+197376w_{2}+582464,$$

$$L_{9,4}=-12288w_{1}^{6}w_{2}^{2}-36864w_{1}^{6}w_{2}+40960w_{1}^{5}w_{2}^{3}+100352w_{1}^{5}w_{2}^{2}-162816w_{1}^{5}w_{2}$$

$$-47104w_{1}^{4}w_{2}^{4}+81920w_{1}^{4}w_{2}^{3}+612352w_{1}^{4}w_{2}^{2}+340480w_{1}^{4}w_{2}+20480w_{1}^{3}w_{2}^{5}$$

$$-346112w_{1}^{3}w_{2}^{4}-1741824w_{1}^{3}w_{2}^{3}-498944w_{1}^{3}w_{2}^{2}+1867136w_{1}^{3}w_{2}$$

$$-2048w_{1}^{2}w_{2}^{6}+227328w_{1}^{2}w_{2}^{5}-16384w_{1}^{2}w_{2}^{4}-4208640w_{1}^{2}w_{2}^{3}$$

$$-5454208w_{1}^{2}w_{2}^{2}+478720w_{1}^{2}w_{2}-26624w_{1}w_{2}^{6}+390144w_{1}w_{2}^{5}$$

$$+1668608w_{1}w_{2}^{4}-195072w_{1}w_{2}^{3}-4043136w_{1}w_{2}^{2}-1638720w_{1}w_{2},$$

$$L_{9,5}=-36864w_{1}^{6}w_{2}-73728w_{1}^{6}+56320w_{1}^{5}w_{2}^{2}+49664w_{1}^{5}w_{2}-542208w_{1}^{5}$$

$$-29696w_{1}^{4}w_{2}^{3}+718848w_{1}^{4}w_{2}^{2}+1289216w_{1}^{4}w_{2}-761088w_{1}^{4}+11264w_{1}^{3}w_{2}^{4}$$

$$+59392w_{1}^{3}w_{2}^{3}+542976w_{1}^{3}w_{2}^{2}+2297216w_{1}^{3}w_{2}+1224064w_{1}^{3}-1024w_{1}^{2}w_{2}^{5}$$

$$+178176w_{1}^{2}w_{2}^{4}+386048w_{1}^{2}w_{2}^{3}-1972992w_{1}^{2}w_{2}^{2}-1768896w_{1}^{2}w_{2}$$

$$+1736768w_{1}^{2}-13824w_{1}w_{2}^{5}+340480w_{1}w_{2}^{4}+1141760w_{1}w_{2}^{3}$$

$$-907712w_{1}w_{2}^{2}-3336512w_{1}w_{2}-587648w_{1},$$

$$L_{9,6}=36864w_{1}^{6}w_{2}-56320w_{1}^{5}w_{2}^{2}+143872w_{1}^{5}w_{2}+29696w_{1}^{4}w_{2}^{3}-375808w_{1}^{4}w_{2}^{3}$$

$$-641536w_{1}^{4}w_{2}-11264w_{1}^{3}w_{2}^{4}+296960w_{1}^{3}w_{2}^{3}-237312w_{1}^{3}w_{2}^{2}$$

$$-3067776w_{1}^{3}w_{2}+1024w_{1}^{2}w_{2}^{5}-137728w_{1}^{2}w_{2}^{4}+249856w_{1}^{2}w_{2}^{3}$$

$$+2163584w_{1}^{2}w_{2}^{2}-2037312w_{1}^{2}w_{2}+27136w_{1}w_{2}^{5}-448512w_{1}w_{2}^{4}$$

$$-1246464w_{1}w_{2}^{3}+2756416w_{1}w_{2}^{2}+2600512w_{1}w_{2}+91648w_{2}^{5}-167808w_{2}^{4}$$

$$-1468672w_{2}^{3}-243264w_{2}^{2}+1742208w_{2},$$

$$L_{10,1}=4096w_{1}^{6}w_{2}^{2}+45056w_{1}^{6}w_{2}+77824w_{1}^{6}-20480w_{1}^{5}w_{2}^{3}-146432w_{1}^{5}w_{2}^{2}$$

$$-14438w_{1}^{5}w_{2}+243200w_{1}^{5}+32768w_{1}^{4}w_{2}^{4}+119808w_{1}^{4}w_{2}^{3}-445440w_{1}^{4}w_{2}^{2}$$

$$-1295360w_{1}^{4}w_{2}-298496w_{1}^{4}-20480w_{1}^{3}w_{2}^{5}+62464w_{1}^{3}w_{2}^{4}+995328w_{1}^{3}w_{2}^{3}$$

$$+1866496w_{1}^{3}w_{2}^{2}-46080w_{1}^{3}w_{2}-897984w_{1}^{3}+4096w_{1}^{2}w_{2}^{6}-80896w_{1}^{2}w_{2}^{5}$$

$$-396288w_{1}^{2}w_{2}^{4}+428544w_{1}^{2}w_{2}^{3}+3323776w_{1}^{2}w_{2}^{2}+3217984w_{1}^{2}w_{2}+$$

$$428544w_{1}^{2}w_{2}^{3}+3323776w_{1}^{4}w_{2}^{2}+3217984w_{1}^{2}w_{2}+372160,$$

$$L_{10,2}=4096w_{1}^{6}w_{2}^{2}-20480w_{1}^{5}w_{2}^{3}-80896w_{1}^{5}w_{2}^{2}+32768w_{1}^{4}w_{2}^{4}+62464w_{1}^{4}w_{2}^{3}$$

$$-396288w_{1}^{4}w_{2}^{2}-20480w_{1}^{3}w_{2}^{5}+119808w_{1}^{3}w_{2}^{4}+995328w_{1}^{3}w_{2}^{3}$$

$$+428544w_{1}^{3}w_{2}^{2}+4096w_{1}^{2}w_{2}^{6}-146432w_{1}^{2}w_{2}^{5}-445440w_{1}^{2}w_{2}^{4}$$

$$+1866496w_{1}^{2}w_{2}^{3}+3323776w_{1}^{2}w_{2}^{2}+45056w_{1}w_{2}^{6}-144384w_{1}w_{2}^{5}$$

$$-1295360w_{1}w_{2}^{4}-46080w_{1}w_{2}^{3}+3217984w_{1}w_{2}^{2}+77824w_{2}^{6}+243200w_{2}^{5}$$

$$-298496w_{2}^{4}-897984w_{2}^{3}+327160w_{2}^{2},$$

$$L_{10,3}=77824w_{1}^{6}+5120w_{1}^{5}w_{2}+354816w_{1}^{5}+9216w_{1}^{4}w_{2}^{2}+186368w_{1}^{4}w_{2}$$

$$-147456w_{1}^{4}-6144w_{1}^{3}w_{2}^{3}+96256w_{1}^{3}w_{2}^{2}+913152w_{1}^{3}w_{2}-2051584w_{1}^{3}$$

$$+1024w_{1}^{2}w_{2}^{4}-42240w_{1}^{2}w_{2}^{3}-33920w_{1}^{2}w_{2}^{2}+1543360w_{1}^{2}w_{2}-1534624w_{1}^{2}$$

$$+572032w_{1}w_{2}+1075520w_{1}+34304w_{2}^{4}-52288w_{2}^{3}-512608w_{2}^{2}$$

$$-393920w_{2}+23040w_{1}w_{2}^{4}-171520w_{1}w_{2}^{3}-680448w_{1}w_{2}^{2}+734720,$$

$$L_{10,4}=-8192w_{1}^{6}w_{2}^{2}-45056w_{1}^{6}w_{2}+40960w_{1}^{5}w_{2}^{3}+227328w_{1}^{5}w_{2}^{2}+96256w_{1}^{5}w_{2}$$

$$-182272w_{1}^{4}w_{2}^{3}+634880w_{1}^{4}w_{2}^{2}+1434112w_{1}^{4}w_{2}+40960w_{1}^{3}w_{2}^{5}$$

$-182272w_{1}^{3}w_{2}^{4}-2537472w_{1}^{3}w_{2}^{3}-2888192w_{1}^{3}w_{2}^{2}+1839104w_{1}^{3}w_{2}$

$-8192w_{1}^{2}w_{2}^{6}+227328w_{1}^{2}w_{2}^{5}+634880w_{1}^{2}w_{2}^{4}-2888192w_{1}^{2}w_{2}^{3}-$

$7538688w_{1}^{2}w_{2}^{2}-1982080w_{1}^{2}w_{2}-65536w_{1}^{4}w_{2}^{4}-45056w_{1}w_{2}^{6}+$

$$96256w_{1}w_{2}^{5}+1434112w_{1}w_{2}^{4}+1839104w_{1}w_{2}^{3}-1982080w_{1}w_{2}^{2}$$

$$-2194560w_{1}w_{2},$$

$$L_{10,5}=-45056w_{1}^{6}w_{2}-155648w_{1}^{6}+61440w_{1}^{5}w_{2}^{2}+123904w_{1}^{5}w_{2}-598016w_{1}^{5}$$

$$-34816w_{1}^{4}w_{2}^{3}+391168w_{1}^{4}w_{2}^{2}+1461248w_{1}^{4}w_{2}+480256w_{1}^{4}+2897280w_{1}^{3}$$

$$-4096w_{1}^{2}w_{2}^{5}+350208w_{1}^{2}w_{2}^{4}+1333760w_{1}^{2}w_{2}^{3}-1333760w_{1}^{2}w_{2}^{3}$$

$$-1315840w_{1}^{2}w_{2}^{2}-4642176w_{1}^{2}w_{2}+649856w_{1}^{2}-15360w_{1}w_{2}^{5}$$

$$+329216w_{1}w_{2}^{4}+1518848w_{1}w_{2}^{3}+799616w_{1}w_{2}^{2}-2757376w_{1}w_{2}$$

$$-1469440w_{1},$$

$$L_{10,6}=45056w_{1}^{6}w_{2}-61440w_{1}^{5}w_{2}^{2}-80896w_{1}^{5}w_{2}+34816w_{1}^{4}w_{2}^{3}-192512w_{1}^{4}w_{2}^{2}$$

$$-1763328w_{1}^{4}w_{2}-22528w_{1}^{3}w_{2}^{4}+165888w_{1}^{3}w_{2}^{3}+697344w_{1}^{3}w_{2}^{2}$$

$$-3357952w_{1}^{3}w_{2}+4096w_{1}^{2}w_{2}^{5}-135168w_{1}^{2}w_{2}^{4}-602112w_{1}^{2}w_{2}^{3}$$

$$+2206976w_{1}^{2}w_{2}^{2}+1182464w_{1}^{2}w_{2}+68608w_{1}w_{2}^{5}-304640w_{1}w_{2}^{4}$$

$$+188288w_{1}w_{2}^{2}+4951936w_{1}w_{2}-2227200w_{1}w_{2}^{3}+111616w_{2}^{5}+116736w_{2}^{4}$$

$$-1101312w_{2}^{3}-1394176w_{2}^{2}+1469440w_{2},$$

$$L_{11,1}=1024w_{1}^{6}w_{2}^{2}+26624w_{1}^{6}w_{2}+80896w_{1}^{6}-10240w_{1}^{5}w_{2}^{3}-126976w_{1}^{5}w_{2}^{2}$$

$$+22528w_{1}^{5}+23552w_{1}^{4}w_{2}^{4}+184320w_{1}^{4}w_{2}^{3}+92928w_{1}^{4}w_{2}^{2}-937728w_{1}^{4}w_{2}$$

$$-848704w_{1}^{4}-20480w_{1}^{3}w_{2}^{5}-67584w_{1}^{3}w_{2}^{4}+477184w_{1}^{3}w_{2}^{3}+2134528w_{1}^{3}w_{2}^{2}$$

$+1629568w_{1}^{3}w_{2}-521536w_{1}^{3}+6144w_{1}^{2}w_{2}^{6}-16384w_{1}^{2}w_{2}^{5}-332288w_{1}^{2}w_{2}^{4}$ $-485376w_{1}^{2}w_{2}^{3}+1419968w_{1}^{2}w_{2}^{2}+2942272w_{1}^{2}w_{2}+922848w_{1}^{2}-$

$$295936w_{1}^{5}w_{2},$$

$$L_{11,2}=1024w_{1}^{6}w_{2}^{2}-10240w_{1}^{5}w_{2}^{3}-100352w_{1}^{5}w_{2}^{2}+23552w_{1}^{4}w_{2}^{4}+161792w_{1}^{4}w_{2}^{3}$$

$$-77824w_{1}^{4}w_{2}^{2}-20480w_{1}^{3}w_{2}^{5}-14336w_{1}^{3}w_{2}^{4}+902144w_{1}^{3}w_{2}^{3}$$

$$+1737216w_{1}^{3}w_{2}^{2}+6144w_{1}^{2}w_{2}^{6}-83968w_{1}^{2}w_{2}^{5}-628224w_{1}^{2}w_{2}^{4}$$

$$+536200w_{1}^{2}w_{2}^{3}+3914752w_{1}^{2}w_{2}^{2}+36864w_{1}w_{2}^{6}-7168w_{1}w_{2}^{5}$$

$$-791552w_{1}w_{2}^{4}-1061248w_{1}w_{2}^{3}+1875392w_{1}w_{2}^{2}+33792w_{2}^{6}+21936w_{2}^{5}$$

$$+272384w_{2}^{4}-419136w_{2}^{3}-231712w_{2}^{2},$$

$$L_{11,3}=80896w_{1}^{6}+107520w_{1}^{5}w_{2}+114176w_{1}^{5}+39424w_{1}^{4}w_{2}^{2}+629760w_{1}^{4}w_{2}$$

$$-990848w_{1}^{4}-9216w_{1}^{3}w_{2}^{3}+261632w_{1}^{3}w_{2}^{2}+1161984w_{1}^{3}w_{2}-2108288w_{1}^{3}$$

$$+4096w_{1}^{2}w_{2}^{4}-15360w_{1}^{2}w_{2}^{3}+174912w_{1}^{2}w_{2}^{2}+385856w_{1}^{2}w_{2}+153008w_{1}^{2}$$

$$+52736w_{1}w_{2}^{4}-42496w_{1}w_{2}^{3}-567616w_{1}w_{2}^{2}-1012768w_{1}w_{2}+1939584w_{1}$$

$$+3072w_{2}^{5}+36096w_{2}^{4}+19456w_{2}^{3}-333200w_{2}^{2}-666880w_{2}+582464,$$

$$L_{11,4}=-2048w_{1}^{6}w_{2}^{2}-26624w_{1}^{6}w_{2}+20480w_{1}^{5}w_{2}^{3}+227328w_{1}^{5}w_{2}^{2}+390144w_{1}^{5}w_{2}$$

$$-47104w_{1}^{4}w_{2}^{4}-346112w_{1}^{4}w_{2}^{3}-16384w_{1}^{4}w_{2}^{2}+1668608w_{1}^{4}w_{2}$$

$$+40960w_{1}^{3}w_{2}^{5}+81920w_{1}^{3}w_{2}^{4}-1741824w_{1}^{3}w_{2}^{3}-4208640w_{1}^{3}w_{2}^{2}$$

$$-195072w_{1}^{3}w_{2}-12288w_{1}^{2}w_{2}^{6}+100352w_{1}^{2}w_{2}^{5} +612352w_{1}^{2}w_{2}^{4}$$

$-498944w_{1}^{2}w_{2}^{3}-5454208w_{1}^{2}w_{2}^{2}-4043136w_{1}^{2}w_{2}-36864w_{1}w_{2}^{6}-$

$$-162816w_{1}w_{2}^{5}+340480w_{1}w_{2}^{4}+1867136w_{1}w_{2}^{3}+478720w_{1}w_{2}^{2}$$

$$-1638720w_{1}w_{2},$$

$$L_{11,5}=-26624w_{1}^{6}w_{2}-161792w_{1}^{6}+25600w_{1}^{5}w_{2}^{2}+174592w_{1}^{5}w_{2}-136704w_{1}^{5}$$

$$-11264w_{1}^{4}w_{2}^{3}-31744w_{1}^{4}w_{2}^{2}+655360w_{1}^{4}w_{2}+1865216w_{1}^{4}+23552w_{1}^{4}w_{2}^{4}$$

$$+490496w_{1}^{3}w_{2}^{3}-310272w_{1}^{3}w_{2}^{2}-1817600w_{1}^{3}w_{2}+2511744w_{1}^{3}$$

$$-11264w_{1}^{2}w_{2}^{5}+428032w_{1}^{2}w_{2}^{4}+1707008w_{1}^{2}w_{2}^{3}+450688w_{1}^{2}w_{2}^{2}$$

$$-4597824w_{1}^{2}w_{2}-1602432w_{1}^{2}+18944w_{1}w_{2}^{5}+301056w_{1}w_{2}^{4}$$

$$+1200640w_{1}w_{2}^{3}+1558592w_{1}w_{2}^{2}-961792w_{1}w_{2}-1742208w_{1},$$

$$L_{11,6}=26624w_{1}^{6}w_{2}-25600w_{1}^{5}w_{2}^{2}-376320w_{1}^{5}w_{2}+11264w_{1}^{4}w_{2}^{3}-6144w_{1}^{4}w_{2}^{2}$$

$$-2009088w_{1}^{4}w_{2}-23552w_{1}^{3}w_{2}^{4}-121856w_{1}^{3}w_{2}^{3}+348160w_{1}^{3}w_{2}^{2}$$

$$-946688w_{1}^{3}w_{2}+11264w_{1}^{2}w_{2}^{5}-74752w_{1}^{2}w_{2}^{4}-1170432w_{1}^{2}w_{2}^{3}$$

$$-402304w_{1}^{2}w_{2}^{2}+4950848w_{1}^{2}w_{2}+127488w_{1}w_{2}^{5}-46592w_{1}w_{2}^{4}$$

$$-2041856w_{1}w_{2}^{3}-2460608w_{1}w_{2}^{2}+4975232w_{1}w_{2}+6144w_{2}^{6}$$

$+103936w_{2}^{5}+216320w_{2}^{4}-385792w_{2}^{3}-1273344w_{2}^{2}+587648w_{2},$

$$L_{12,1}=6144w_{1}^{6}w_{2}^{2}+30720w_{1}^{6}-2048w_{1}^{5}w_{2}^{3}-50176w_{1}^{5}w_{2}^{2}-237568w_{1}^{5}w_{2}$$

$$-180736w_{1}^{5}+8192w_{1}^{4}w_{2}^{4}+120832w_{1}^{4}w_{2}^{3}+391168w_{1}^{4}w_{2}^{2}+36608w_{1}^{4}w_{2}$$

$$-628736w_{1}^{4}-10240w_{1}^{3}w_{2}^{5}-96256w_{1}^{3}w_{2}^{4}-79360w_{1}^{3}w_{2}^{3}+1103104w_{1}^{3}w_{2}^{2}$$

$+2140672w_{1}^{3}w_{2}+366272w_{1}^{3}+4096w_{1}^{2}w_{2}^{6}+19456w_{1}^{2}w_{2}^{5}-95744w_{1}^{2}w_{2}^{4}$ $-456704w_{1}^{2}w_{2}^{3}+41728w_{1}^{2}w_{2}^{2}+1472320w_{1}^{2}w_{2}+896640w_{1}^{2},$

$$L_{12,2}=-2048w_{1}^{5}w_{2}^{3}-56320w_{1}^{5}w_{2}^{2}+8192w_{1}^{4}w_{2}^{4}+125952w_{1}^{4}w_{2}^{3}+326656w_{1}^{4}w_{2}^{2}$$

$$-10240w_{1}^{3}w_{2}^{5}-56320w_{1}^{3}w_{2}^{4}+424448w_{1}^{3}w_{2}^{3}+2117276w_{1}^{3}w_{2}^{2}$$

$$+4096w_{1}^{2}w_{2}^{6}-25600w_{1}^{2}w_{2}^{5}-302592w_{1}^{2}w_{2}^{4}+17408w_{1}^{2}w_{2}^{3}$$

$$+2528768w_{1}^{2}w_{2}^{2}+12288w_{1}w_{2}^{6}-29696w_{1}w_{2}^{5}-159488w_{1}w_{2}^{4}$$

$$-474912w_{1}w_{2}^{3}+105856w_{1}w_{2}^{2}-16384w_{2}^{6}+30720w_{2}^{5}+337920w_{2}^{4}$$

$$+238976w_{2}^{3}-444672w_{2}^{2},$$

$$L_{12,3}=43008w_{1}^{6}+173056w_{1}^{5}w_{2}-156160w_{1}^{5}+156672w_{1}^{4}w_{2}^{2}+549888w_{1}^{4}w_{2}$$

$$-1102848w_{1}^{4}+19456w_{1}^{3}w_{2}^{3}+604160w_{1}^{3}w_{2}^{2}+54272w_{1}^{3}w_{2}-767488w_{1}^{3}$$

$$+9216w_{1}^{2}w_{2}^{4}+141312w_{1}^{2}w_{2}^{3}+356352w_{1}^{2}w_{2}^{2}-1381760w_{1}^{2}w_{2}$$

$$+1669440w_{1}^{2}+1024w_{1}w_{2}^{5}+81920w_{1}w_{2}^{4}+213248w_{1}w_{2}^{3}-524672w_{1}w_{2}^{2}$$

$$-1569472w_{1}w_{2}+1720256w_{1}+13824w_{2}^{5}+55808w_{2}^{4}+52352w_{2}^{3}$$

$$-288512w_{2}^{2}-375104w_{2}+250752,$$

$$L_{12,4}=-6144w_{1}^{6}w_{2}+4096w_{1}^{5}w_{2}^{3}+106496w_{1}^{5}w_{2}^{2}\mp353280w_{1}^{5}w_{2}-16384w_{1}^{4}w_{2}^{4}$$

$$-246784w_{1}^{4}w_{2}^{3}-634880w_{1}^{4}w_{2}^{2}+535808w_{1}^{4}w_{2}+20480w_{1}^{3}w_{2}^{5}$$

$$+152576w_{1}^{3}w_{2}^{4}-432128w_{1}^{3}w_{2}^{3}-2983680w_{1}^{3}w_{2}^{2}-2287104w_{1}^{3}w_{2}$$

$$-8192w_{1}^{2}w_{2}^{6}+6144w_{1}^{2}w_{2}^{5} +165888w_{1}^{2}w_{2}^{4}+446464w_{1}^{2}w_{2}^{3}$$

$$-1625344w_{1}^{2}w_{2}^{2}-3654848w_{1}^{2}w_{2}-12288w_{1}w_{2}^{6}-189440w_{1}w_{2}^{5}$$

$$-390912w_{1}w_{2}^{4}+512896w_{1}w_{2}^{3}+1134272w_{1}w_{2}^{2}-551040w_{1}w_{2},$$

$$L_{12,5}=-6144w_{1}^{6}w_{2}-73728w_{1}^{6}-2048w_{1}^{5}w_{2}^{2}+124928w_{1}^{5}w_{2}+343040w_{1}^{5}$$

$$+10240w_{1}^{4}w_{2}^{3}-45056w_{1}^{4}w_{2}^{2}-162816w_{1}^{4}w_{2}+1717248w_{1}^{4}+22528w_{1}^{3}w_{2}^{4}$$

$$+374784w_{1}^{3}w_{2}^{3}+183552w_{1}^{3}w_{2}^{2}-2059008w_{1}^{3}w_{2}+184128w_{1}^{3}$$

$$-22528w_{1}^{2}w_{2}^{5}+299008w_{1}^{2}w_{2}^{4}+1345024w_{1}^{2}w_{2}^{3}+1323008w_{1}^{2}w_{2}^{2}$$

$$-1955904w_{1}^{2}w_{2}-2824096w_{1}^{2}-2048w_{1}w_{2}^{6}+57344w_{1}w_{2}^{5}+305664w_{1}w_{2}^{4}$$

$$+719872w_{1}w_{2}^{3}+1038912w_{1}w_{2}^{2}+276256w_{1}w_{2}-1172160w_{1},$$

$$L_{12,6}=6144w_{1}^{6}w_{2}+2048w_{1}^{5}w_{2}^{2}-413696w_{1}^{5}w_{2}-10240w_{1}^{4}w_{2}^{3}-196608w_{1}^{4}w_{2}^{2}$$

$$-969728w_{1}^{4}w_{2}-22528w_{1}^{3}w_{2}^{4}-307200w_{1}^{3}w_{2}^{3}-1046016w_{1}^{3}w_{2}^{2}$$

$$+1996032w_{1}^{3}w_{2}+22528w_{1}^{2}w_{2}^{5}-65536w_{1}^{2}w_{2}^{4}-1357824w_{1}^{2}w_{2}^{3}$$

$$-2272896w_{1}^{2}w_{2}^{2}+5683648w_{1}^{2}w_{2}+2048w_{1}w_{2}^{6}+151552w_{1}w_{2}^{5}$$

$$+32256w_{1}w_{2}^{4}-1578240w_{1}w_{2}^{3}-2040192w_{1}w_{2}^{2}+2679904w_{1}w_{2}$$

$$+26624w_{2}^{6}+100352w_{2}^{5}+73728w_{2}^{4}-141568w_{2}^{3}-185440w_{2}^{2}-169152,$$

$$L_{13,1}=6144w_{1}^{6}w_{2}+15360w_{1}^{6}-2048w_{1}^{5}w_{2}^{3}-32768w_{1}^{5}w_{2}^{2}-132608w_{1}^{5}w_{2}$$

$$-201728w_{1}^{5}+5120w_{1}^{4}w_{2}^{4}+60416w_{1}^{4}w_{2}^{3}+149248w_{1}^{4}w_{2}^{2}+79104w_{1}^{4}w_{2}$$

$$-346496w_{1}^{4}-4096w_{1}^{3}w_{2}^{5}-16384w_{1}^{3}w_{2}^{4}+142336w_{1}^{3}w_{2}^{3}+882944w_{1}^{3}w_{2}^{2}$$

$+1575168w_{1}^{3}w_{2}+550016w_{1}^{3}+1024w_{1}^{2}w_{2}^{6}-17408w_{1}^{2}w_{2}^{5}-158720w_{1}^{2}w_{2}^{4}$ $-221696w_{1}^{2}w_{2}^{3}+523136w_{1}^{2}w_{2}^{2}+1267584w_{1}^{2}w_{2}+557504w_{1}^{2},$

$$L_{13,2}=-2048w_{1}^{5}w_{2}^{3}-44032w_{1}^{5}w_{2}^{2}+5120w_{1}^{4}w_{2}^{4}+65536w_{1}^{4}w_{2}^{3}+222976w_{1}^{4}w_{2}^{2}$$

$$-4096w_{1}^{3}w_{2}^{5}+16384w_{1}^{3}w_{2}^{4}+516096w_{1}^{3}w_{2}^{3}+1225472w_{1}^{3}w_{2}^{2}+1024w_{1}^{2}w_{2}^{6}$$

$$-37888w_{1}^{2}w_{2}^{5}-56576w_{1}^{2}w_{2}^{4}+1068544w_{1}^{2}w_{2}^{3}+1342144w_{1}^{2}w_{2}^{2}$$

$$-109568w_{1}w_{2}^{5}-147200w_{1}w_{2}^{4}+783616w_{1}w_{2}^{3}+745200w_{1}w_{2}^{2}-31744w_{2}^{6}$$

$$-108032w_{2}^{5}+25792w_{2}^{4}+226880w_{2}^{3}-352448w_{2}^{2},$$

$$L_{13,3}=15360w_{1}^{6}+126976w_{1}^{5}w_{2}-155648w_{1}^{5}+244736w_{1}^{4}w_{2}^{2}+186368w_{1}^{4}w_{2}$$

$$-652288w_{1}^{4}+119808w_{1}^{3}w_{2}^{3}+583168w_{1}^{3}w_{2}^{2}-485888w_{1}^{3}w_{2}-206336w_{1}^{3}$$

$$+30208w_{1}^{2}w_{2}^{4}+367616w_{1}^{2}w_{2}^{3}-203456w_{1}^{2}w_{2}^{2}-1003840w_{1}^{2}w_{2}$$

$$+1241008w_{1}^{2}+4096w_{1}w_{2}^{5}+152064w_{1}w_{2}^{4}+289280w_{1}w_{2}^{3}$$

$$-451872w_{1}w_{2}+1185664w_{1}+24576w_{2}^{5}+143488w_{2}^{4}+13184w_{2}^{3}$$

$$-571792w_{2}^{2}-15872w_{2}+158016,$$

$$L_{13,4}=-6144w_{1}^{6}w_{2}+4096w_{1}^{5}w_{2}^{3}+76800w_{1}^{5}w_{2}^{2}+235008w_{1}^{5}w_{2}-10240w_{1}^{4}w_{2}^{4}$$

$$-125952w_{1}^{4}w_{2}^{3}-242688w_{1}^{4}w_{2}^{2}+21760w_{1}^{4}w_{2}+8192w_{1}^{3}w_{2}^{5}-$$

$$721408w_{1}^{3}w_{2}^{3}-1830912w_{1}^{3}w_{2}^{2}-1955968w_{1}^{3}w_{2}-2048w_{1}^{2}w_{2}^{6}$$

$$+55296w_{1}^{2}w_{2}^{5} +82944w_{1}^{2}w_{2}^{4}-772096w_{1}^{2}w_{2}^{3}-1742336w_{1}^{2}w_{2}^{2}$$

$$-2228672w_{1}^{2}w_{2}-32768w_{1}w_{2}^{5}-79872w_{1}w_{2}^{4} +106752w_{1}w_{2}^{3}$$

$$+224960w_{1}w_{2}^{2}-427008w_{1}w_{2},$$

$$L_{13,5}=-6144w_{1}^{6}w_{2}-30720w_{1}^{6}-11264w_{1}^{5}w_{2}^{2}+2560w_{1}^{5}w_{2}+357376w_{1}^{5}$$

$$+14336w_{1}^{4}w_{2}^{3}-7680w_{1}^{4}w_{2}^{2}+37120w_{1}^{4}w_{2}+1017984w_{1}^{4}+36864w_{1}^{3}w_{2}^{4}$$

$$+448000w_{1}^{3}w_{2}^{3}+124160w_{1}^{3}w_{2}^{2}-944384w_{1}^{3}w_{2}-496192w_{1}^{3}-25600w_{1}^{2}w_{2}^{5}$$

$$+260096w_{1}^{2}w_{2}^{4}+1403648w_{1}^{2}w_{2}^{3}+571648w_{1}^{2}w_{2}^{2}-1733184w_{1}^{2}w_{2}$$

$$-2021728w_{1}^{2}-8192w_{1}w_{2}^{6}+6656w_{1}w_{2}^{5}+383744w_{1}w_{2}^{4}+1077120w_{1}w_{2}^{3}$$

$$+685504w_{1}w_{2}^{2}-370720w_{1}w_{2}-771008w_{1},$$

$$L_{13,6}=6144w_{1}^{6}w_{2}+11264w_{1}^{5}w_{2}^{2}-231936w_{1}^{5}w_{2}-14336w_{1}^{4}w_{2}^{3}-366592w_{1}^{4}w_{2}^{2}$$

$$-312064w_{1}^{4}w_{2}-36864w_{1}^{3}w_{2}^{4}-508928w_{1}^{3}w_{2}^{3}-1013248w_{1}^{3}w_{2}^{2}$$

$$+1689984w_{1}^{3}w_{2}+25600w_{1}^{2}w_{2}^{5}-157696w_{1}^{2}w_{2}^{4}-1840128w_{1}^{2}w_{2}^{3}$$

$$-505856w_{1}^{2}w_{2}^{2}+3580416w_{1}^{2}w_{2}+8192w_{1}w_{2}^{6}+143872w_{1}w_{2}^{5}$$

$$-190720w_{1}w_{2}^{4}-2034432w_{1}w_{2}^{3}+350080w_{1}w_{2}^{2}+1776544w_{1}w_{2}$$

$$+38912w_{2}^{6}+159232w_{2}^{5}-83456w_{2}^{4}-587328w_{2}^{3}+232288w_{2}^{2}$$

$$-138944w_{2},$$

$$L_{14,1}=3072w_{1}^{4}w_{2}^{3}-89600w_{1}^{5}-14336w_{1}^{5}w_{2}+62976w_{1}^{4}w_{2}^{2}+217088w_{1}^{4}w_{2}$$

$$+94464w_{1}^{4}-6144w_{1}^{3}w_{2}^{4}-62464w_{1}^{3}w_{2}^{3}-89088w_{1}^{3}w_{2}^{2}+319744w_{1}^{3}w_{2}$$

$$+557120w_{1}^{3}+3072w_{1}^{2}w_{2}^{5}+13824w_{1}^{2}w_{2}^{4}-1280w_{1}^{2}w_{2}^{3}-74880w_{1}^{2}w_{2}^{2}$$

$$+34496w_{1}^{2}w_{2}+265728w_{1}^{2},$$

$$L_{14,2}=5120w_{1}^{4}w_{2}^{3}+198144w_{1}^{4}w_{2}^{2}+12288w_{1}^{3}w_{2}^{4}+246784w_{1}^{3}w_{2}^{3}+326400w_{1}^{3}w_{2}^{2}$$

$$-5120w_{1}^{2}w_{2}^{5}+128512w_{1}^{2}w_{2}^{4}+615936w_{1}^{2}w_{2}^{3}-356736w_{1}^{2}w_{2}^{2}-12288w_{1}w_{2}^{6}$$

$$-32768w_{1}w_{2}^{5}+205824w_{1}w_{2}^{4}+486656w_{1}w_{2}^{3}-604608w_{1}w_{2}^{2}-36864w_{2}^{6}$$

$$-108032w_{2}^{5}+32000w_{2}^{4}+182464w_{2}^{3}-49920w_{2}^{2},$$

$$L_{14,3}=27648w_{1}^{5}w_{2}-89600w_{1}^{5}+166912w_{1}^{4}w_{2}^{2}-246784w_{1}^{4}w_{2}-56532w_{1}^{4}$$

$$+210944w_{1}^{3}w_{2}^{3}+130304w_{1}^{3}w_{2}^{2}-912384w_{1}^{3}w_{2}+768064w_{1}^{3}$$

$$+84992w_{1}^{3}w_{2}^{4}+463872w_{1}^{2}w_{2}^{3}-646016w_{1}^{3}w_{2}^{2}-779776w_{1}^{2}w_{2}$$

$$+1008992w_{1}^{2}+13312w_{1}w_{2}^{5}+204800w_{1}w_{2}^{4}+226304w_{1}w_{2}^{3}+37376w_{2}^{5}$$

$$+152576w_{2}^{4}-5248w_{2}^{3}-257248w_{2}^{2}+134336w_{2}-40192,$$

$$L_{14,4}=41984w_{1}^{5}w_{2}-8192w_{1}^{4}w_{2}^{3}-94208w_{1}^{4}w_{2}^{2}-470528w_{1}^{4}w_{2}-6144w_{1}^{3}w_{2}^{4}$$

$$+27648w_{1}^{3}w_{2}^{3}-115712w_{1}^{3}w_{2}^{2}-1211904w_{1}^{3}w_{2}+2048w_{1}^{2}w_{2}^{5}-$$

$$49152w_{1}^{2}w_{2}^{4}-10832w_{1}^{2}w_{2}^{3}+57344w_{1}^{2}w_{2}^{2}-266368w_{1}^{2}w_{2}+12288w_{1}w_{2}^{6}$$

$$+1024w_{1}w_{2}^{5}-195072w_{1}w_{2}^{4}-397824w_{1}w_{2}^{3}-50560w_{1}w_{2}^{2}+376576w_{1}w_{2},$$

$$L_{14,5}=-13312w_{1}^{5}w_{2}+179200w_{1}^{5}+2048w_{1}^{4}w_{2}^{3}-31744w_{1}^{4}w_{2}^{2}+50688w_{1}^{4}w_{2}$$

$$-88832w_{1}^{4}+18432w_{1}^{3}w_{2}^{4}+94208w_{1}^{3}w_{2}^{3}+214528w_{1}^{3}w_{2}^{2}+250880w_{1}^{3}w_{2}$$

$$-1352832w_{1}^{3}-8192w_{1}^{2}w_{2}^{5}+30720w_{1}^{2}w_{2}^{4}+272384w_{1}^{2}w_{2}^{3}+577280w_{1}^{2}w_{2}^{2}$$

$$+297088w_{1}^{2}w_{2}-1165696w_{1}^{2}-12288w_{1}w_{2}^{6}-7168w_{1}w_{2}^{5}+148992w_{1}w_{2}^{4}$$

$$+324864w_{1}w_{2}^{3}+257152w_{1}w_{2}^{2}+39040w_{1}w_{2}-77440w_{1},$$

$$L_{14,6}=-41984w_{1}^{5}w_{2}-2048w_{1}^{4}w_{2}^{3}-302080w_{1}^{4}w_{2}^{2}+449536w_{1}^{4}w_{2}-18432w_{1}^{3}w_{2}^{4}$$

$$-517120w_{1}^{3}w_{2}^{3}-466432w_{1}^{3}w_{2}^{2}+1550080w_{1}^{3}w_{2}+98192w_{1}^{2}w_{2}^{5}$$

$$-208896w_{1}^{2}w_{2}^{4}-1229312w_{1}^{2}w_{2}^{3}+466944w_{1}^{2}w_{2}^{2}+826624w_{1}^{2}w_{2}$$

$$+12288w_{1}w_{2}^{6}+25600w_{1}w_{2}^{5}-367616w_{1}w_{2}^{4}-703488w_{1}w_{2}^{3}$$

$$+981376w_{1}w_{2}^{2}-605312w_{1}w_{2}+36864w_{2}^{6}+73728w_{2}^{5}-146176w_{2}^{4}$$

$$-25600w_{2}^{3}+441472w_{2}^{2}-238208w_{2},$$

$$L_{15,1}=6400w_{1}^{4}w_{2}^{2}-15360w_{1}^{5}+56832w_{1}^{4}w_{2}+122432w_{1}^{4}-6144w_{1}^{3}w_{2}^{3}$$

$$-37632w_{1}^{3}w_{2}^{2}+16768w_{1}^{3}w_{2}+221120w_{1}^{3}-256w_{1}^{2}w_{2}^{2}+2560w_{1}^{2}w_{2}^{3}$$

$$+5440w_{1}^{2}w_{2}^{2}+8000w_{1}^{2}w_{2}+60480w_{1}^{2},$$

$$L_{15,2}=38400w_{1}^{4}w_{2}^{2}+5120w_{1}^{3}w_{2}^{4}+168448w_{1}^{3}w_{2}^{3}-52224w_{1}^{3}w_{2}^{2}+3072w_{1}^{2}w_{2}^{5}$$

$$+151296w_{1}^{2}w_{2}^{4}+331008w_{1}^{2}w_{2}^{3}-319488w_{1}^{2}w_{2}^{2}-8192w_{1}w_{2}^{6}$$

$$+4608w_{1}w_{2}^{5}+219392w_{1}w_{2}^{4}+136320w_{1}w_{2}^{3}-194496w_{1}w_{2}^{2}-13312w_{2}^{6}$$

$$-42496w_{2}^{5}-6272w_{2}^{4}-21824w_{2}^{3}+41280w_{2}^{2},$$

$$L_{15,3}=-15360w_{1}^{5}+42240w_{1}^{4}w_{2}^{2}-154880w_{1}^{4}w_{2}+107072w_{1}^{4}+148480w_{1}^{3}w_{2}^{3}$$

$$-184832w_{1}^{3}w_{2}^{2}-288128w_{1}^{3}w_{2}+397248w_{1}^{3}+124928w_{1}^{2}w_{2}^{4}$$

$$+193792w_{1}^{2}w_{2}^{3}-658816w_{1}^{2}w_{2}^{2}+54656w_{1}^{2}w_{2}+285008w_{1}^{2}$$

$$+32768w_{1}w_{2}^{5}+218112w_{1}w_{2}^{4}-65536w_{1}w_{2}^{3}-527616w_{1}w_{2}^{2}+257696w_{1}w_{2}$$

$$-33792w_{1}+1024w_{2}^{6}+43008w_{2}^{5}+119808w_{2}^{4}-51648w_{2}^{3}-102384w_{2}^{2}$$

$$+81792w_{2}-24640,$$

$$L_{15,4}=-2560w_{1}^{4}w_{2}^{2}-211712w_{1}^{4}w_{2}-5120w_{1}^{3}w_{2}^{4}-13824w_{1}^{3}w_{2}^{3}-100096w_{1}^{3}w_{2}^{2}$$

$$-252672w_{1}^{3}w_{2}-3072w_{1}^{2}w_{2}^{5}-23552w_{1}^{2}w_{2}^{4}-120320w_{1}^{2}w_{2}^{3}$$

$-217472w_{1}^{2}w_{2}^{2}+224256w_{1}^{2}w_{2}+8192w_{1}w_{2}^{6} +27136w_{1}w_{2}^{5}-29952w_{1}w_{2}^{4}$ $-192896w_{1}w_{2}^{3}-167936w_{1}w_{2}^{2}+200320w_{1}w_{2},$

$$L_{15,5}=30720w_{1}^{5}-10240w_{1}^{4}w_{2}^{2}+98048w_{1}^{4}w_{2}-229504w_{1}^{4}+5120w_{1}^{3}w_{2}^{4}$$

$$+26112w_{1}^{3}w_{2}^{3}+168960w_{1}^{3}w_{2}^{2}+146304w_{1}^{3}w_{2}-622208w_{1}^{3}+3072w_{1}^{2}w_{2}^{5}$$

$$+24064w_{1}^{2}w_{2}^{4}+126464w_{1}^{2}w_{2}^{3}+321280w_{1}^{2}w_{2}^{2}-72896w_{1}^{2}w_{2}-283712w_{1}^{2}$$

$$-8192w_{1}w_{2}^{6}-27136w_{1}w_{2}^{5}+31488w_{1}w_{2}^{4}+187520w_{1}w_{2}^{3}+162624w_{1}w_{2}^{2}$$

$$-117568w_{1}w_{2}+39680w_{1},$$

$$L_{15,6}=-74240w_{1}^{4}w_{2}^{2}+211712w_{1}^{4}w_{2}-5120w_{1}^{3}w_{2}^{4}-323072w_{1}^{3}w_{2}^{3}$$

$$+205824w_{1}^{3}w_{2}^{2}+377728w_{1}^{3}w_{2}-3072w_{1}^{2}w_{2}^{5}-276480w_{1}^{2}w_{2}^{4}$$

$$-533504w_{1}^{2}w_{2}^{3}+8706564w_{1}^{2}w_{2}^{2}-191808w_{1}^{2}w_{2}+8192w_{1}w_{2}^{6}$$

$$-37376w_{1}w_{2}^{5}-439040w_{1}w_{2}^{4}-69504w_{1}w_{2}^{3}+675648w_{1}w_{2}^{2}$$

$$-450112w_{1}w_{2}+12288w_{2}^{6}-512w_{2}^{5}-113280w_{2}^{4}+90880w_{2}^{3}+123072w_{2}^{2}$$

$$-58880w_{2},$$

$$L_{16,1}=4608w_{1}^{4}w_{2}+45568w_{1}^{4}+1024w_{1}^{3}w_{2}^{3}+512w_{1}^{3}w_{2}^{2}+4736w_{1}^{3}w_{2}+42176w_{1}^{3}$$

$$-1024w_{1}^{2}w_{2}^{4}-4608w_{1}^{2}w_{2}^{3}+2304w_{1}^{2}w_{2}^{2}+18496w_{1}^{2}w_{2}+3712w_{1}^{2},$$

$$L_{16,2}=41472w_{1}^{3}w_{2}^{3}-51456w_{1}^{3}w_{2}^{2}+2048w_{1}^{2}w_{2}^{5}+98304w_{1}^{2}w_{2}^{4}+44288w_{1}^{2}w_{2}^{3}$$

$$-92928w_{1}^{2}w_{2}^{2}-2048w_{1}w_{2}^{6}+29696w_{1}w_{2}^{5}+116736w_{1}w_{2}^{4}-45440w_{1}w_{2}^{3}$$

$$-2112w_{1}w_{2}^{2}+2048w_{2}^{6}+13312w_{2}^{5}-15616w_{2}^{4}-54976w_{2}^{3}+34176w_{2}^{2},$$

$$L_{16,3}=-30720w_{1}^{4}+45568w_{1}^{4}+38400w_{1}^{3}w_{2}^{3}-139008w_{1}^{3}w_{2}^{2}+24448w_{1}^{3}w_{2}$$

$$+96576w_{1}^{3}+84992w_{1}^{2}w_{2}^{4}-40192w_{1}^{2}w_{2}^{3}-286208w_{1}^{2}w_{2}^{2}+187584w_{1}^{2}w_{2}$$

$$+20288w_{1}^{2}+44032w_{1}w_{2}^{5}+121344w_{1}w_{2}^{4}-158336w_{1}w_{2}^{3}-155200w_{1}w_{2}^{2}$$

$$+134464w_{1}w_{2}-39872w_{1}+4096w_{2}^{6}+39424w_{2}^{5}+45312w_{2}^{4}-44096w_{2}^{3}$$

$$-2304w_{2}^{2}+13888w_{2}-4480,$$

$$L_{16,4}=-35328w_{1}^{4}w_{2}-4096w_{1}^{3}w_{2}^{3}-88064w_{1}^{3}w_{2}^{2}+32768w_{1}^{3}w_{2}-2048w_{1}^{2}w_{2}^{5}$$

$$-12288w_{1}^{2}w_{2}^{4}-78848w_{1}^{2}w_{2}^{3}-145408w_{1}^{2}w_{2}^{2}+144256w_{1}^{2}w_{2}+$$

$2048w_{1}w_{2}^{6} +15360w_{1}w_{2}^{5}+11264w_{1}w_{2}^{4}-68608w_{1}w_{2}^{3}-61824w_{1}w_{2}^{2}$ $+55808w_{1}w_{2},$

$$L_{16,5}=26112w_{1}^{4}w_{2}-91136w_{1}^{4}+2048w_{1}^{3}w_{2}^{3}+87040w_{1}^{2}w_{2}^{3}+143872w_{1}^{2}w_{2}^{2}$$

$$-153600w_{1}^{2}w_{2}-8192w_{1}^{2}-2048w_{1}w_{2}^{6}-15360w_{1}w_{2}^{5}-9728w_{1}w_{2}^{4}$$

$$+79360w_{1}w_{2}^{3}+66688w_{1}w_{2}^{2}-67072w_{1}w_{2}+24192w_{1},$$

$$L_{16,6}=35328w_{1}^{4}w_{2}-78848w_{1}^{3}w_{2}^{3}+190976w_{1}^{3}w_{2}^{2}-15104w_{1}^{3}w_{2}-2048w_{1}^{2}w_{2}^{5}$$

$$-184320w_{1}^{2}w_{2}^{4}-7680w_{1}^{2}w_{2}^{3}+378368w_{1}^{2}w_{2}^{2}-195584w_{1}^{2}w_{2}$$

$$-73728w_{1}w_{2}^{5}-239616w_{1}w_{2}^{4}+193280w_{1}w_{2}^{3}+150784w_{1}w_{2}^{2}$$

$$-140032w_{1}w_{2}^{2}-6144w_{2}^{6}-52736w_{2}^{5}-30208w_{2}^{4}+95616w_{2}^{3}$$

$$-26624w_{2}^{2}+2048w_{1}w_{2}^{6} +6272w_{2},$$

$L_{17,1}=6400w_{1}^{4}+1536w_{1}^{3}w_{2}^{2}+9856w_{1}^{3}w_{2}+960w_{1}^{3}-1536w_{1}^{2}w_{2}^{3}-1344w_{1}^{2}w_{2}^{2}$

$$+7424w_{1}^{2}w_{2}-1264w_{1}^{2},$$

$$L_{17,2}=-9216w_{1}^{3}w_{2}^{2}+24832w_{1}^{2}w_{2}^{4}-18432w_{1}^{2}w_{2}^{3}-4032w_{1}^{2}w_{2}^{2}+25600w_{1}w_{2}^{5}$$

$$+20736w_{1}w_{2}^{4}-38784w_{1}w_{2}^{3}+19008w_{1}w_{2}^{2}+6144w_{2}^{6}+20480w_{2}^{5}$$

$$-11968w_{2}^{4}-24000w_{2}^{3}+11664w_{2}^{2},$$

$$L_{17,3}=6400w_{1}^{4}-29440w_{1}^{3}w_{2}^{2}+36352w_{1}^{3}w_{2}+7360w_{1}^{3}+21760w_{1}^{2}w_{2}^{4}$$

$$-59392w_{1}^{2}w_{2}^{3}-44288w_{1}^{2}w_{2}^{2}+67968w_{1}^{2}w_{2}-9168w_{1}^{2}+28672w_{1}w_{2}^{5}$$

$$+22272w_{1}w_{2}^{4}-82816w_{1}w_{2}^{3}-2432w_{1}w_{2}^{2}+22368w_{1}w_{2}-10240w_{1}$$

$$+6144w_{2}^{6}+23552w_{2}^{5}+1088w_{2}^{4}-16960w_{2}^{3}+14768w_{2}^{2}-4864w_{2}$$

$$+896,$$

$$L_{17,4}=-21760w_{1}^{3}w_{2}^{2}+26496w_{1}^{3}w_{2}-3072w_{1}^{2}w_{2}^{4}-39424w_{1}^{2}w_{2}^{3}-29568w_{1}^{2}w_{2}^{2}$$

$$+33792w_{1}^{2}w_{2}+3072w_{1}w_{2}^{5}-3072w_{1}w_{2}^{4}-20480w_{1}w_{2}^{3}-3008w_{1}w_{2}^{2}$$

$$+6816w_{1}w_{2},$$

$$L_{17,5}=-12800w_{1}^{4}+18688w_{1}^{3}w_{2}^{2}-46208w_{1}^{3}w_{2}-8320w_{1}^{3}+3072w_{1}^{2}w_{2}^{4}$$

$$+42496w_{1}^{2}w_{2}^{3}+30720w_{1}^{2}w_{2}^{2}-57536w_{1}^{2}w_{2}+12032w_{1}^{2}-3072w_{1}w_{2}^{5}$$

$$-3072w_{1}w_{2}^{4}+22784w_{1}w_{2}^{3}+8384w_{1}w_{2}^{2}-11840w_{1}w_{2}+4672w_{1},$$

$$L_{17,6}=40192w_{1}^{3}w_{2}^{2}-26496w_{1}^{3}w_{2}-46592w_{1}^{2}w_{2}^{4}-76288w_{1}^{2}w_{2}^{3}+48512w_{1}^{2}w_{2}^{2}$$

$$-51648w_{1}^{2}w_{2}-54272w_{1}w_{2}^{5}-43008w_{1}w_{2}^{4}+119296w_{1}w_{2}^{3}$$

$$-15360w_{1}w_{2}-12288w_{2}^{6}-44032w_{2}^{5}+10880w_{2}^{4}+40192w_{2}^{3}$$

$$-28352w_{2}^{2}-21568w_{1}w_{2}^{2}+8256w_{2},$$

$$L_{18,1}=2560w_{1}^{3}w_{2}-640w_{1}^{3}+256w_{1}^{2}w_{2}^{2}+576w_{1}^{2}w_{2}-1264w_{1}^{2},$$

$$L_{18,2}=-5376w_{1}^{2}w_{2}^{3}-3456w_{1}^{2}w_{2}^{2}+7168w_{1}w_{2}^{5}-4096w_{1}w_{2}^{4}-9216w_{1}w_{2}^{3}$$

$$+5760w_{1}w_{2}^{2}+4096w_{2}^{6}+7680w_{2}^{5}-4096w_{2}^{4}-4800w_{2}^{3}+2016w_{2}^{2},$$

$$L_{18,3}=6400w_{1}^{3}w_{2}-640w_{1}^{3}-14080w_{1}^{2}w_{2}^{3}+4736w_{1}^{2}w_{2}^{2}+7360w_{1}^{2}w_{2}$$

$$+7168w_{1}w_{2}^{5}-6656w_{1}w_{2}^{4}-17408w_{1}w_{2}^{3}+9856w_{1}w_{2}^{2}-2048w_{1}w_{2}$$

$$-704w_{1}+4096w_{2}^{6}+7680w_{2}^{5}-4608w_{2}^{4}-2880w_{2}^{3}+5472w_{2}^{2}-2496w_{2}$$

$$-1760w_{1}^{2}+512,$$

$$L_{18,4}=3840w_{1}^{3}w_{2}-8704w_{1}^{2}w_{2}^{3}+1024w_{1}^{2}w_{2}^{2}+2304w_{1}^{2}w_{2}-2560w_{1}w_{2}^{4}$$

$$-3840w_{1}w_{2}^{3}+2944w_{1}w_{2}^{2}-192w_{1}w_{2},$$

$$L_{18,5}=-8960w_{1}^{3}w_{2}+1280w_{1}^{3}+8704w_{1}^{2}w_{2}^{3}-1536w_{1}^{2}w_{2}^{2}-6016w_{1}^{2}w_{2}$$

$$+1920w_{1}^{2}+2560w_{1}w_{2}^{4}+3840w_{1}w_{2}^{3}-2816w_{1}w_{2}^{2}+960w_{1}w_{2}+64w_{1},$$

$$L_{18,6}=-3840w_{1}^{3}w_{2}-19456w_{1}^{2}w_{2}^{3}-7936w_{1}^{2}w_{2}^{2}-4224w_{1}^{2}w_{2}-14336w_{1}w_{2}^{5}$$

$$+10752w_{1}w_{2}^{4}+26624w_{1}w_{2}^{3}-15744w_{1}w_{2}^{2}+1920w_{1}w_{2}-8192w_{2}^{6}$$

$$-15360w_{2}^{5}+8704w_{2}^{4}+7680w_{2}^{3}-7616w_{2}^{2}+2122w_{2},$$

$$L_{19,1}=256w_{1}^{2}w_{2}^{2}-128w_{1}^{2}w_{2}+16w_{1}^{2},$$

$$L_{19,2}=576w_{1}^{2}w_{2}^{2}-1536w_{1}w_{2}^{4}-768w_{1}w_{2}^{3}+576w_{1}w_{2}^{2}+1024w_{2}^{6}+1024w_{2}^{5}$$

$$-512w_{2}^{4}-384w_{2}^{3}+144w_{2}^{2},$$

$$L_{19,3}=1600w_{1}^{2}w_{2}^{2}-320w_{1}^{2}w_{2}+16w_{1}^{2}-2560w_{1}w_{2}^{4}-1024w_{1}w_{2}^{3}+1728w_{1}w_{2}^{2}$$

$$-800w_{1}w_{2}+64w_{1}+1024w_{2}^{6}-1024w_{2}^{5}-1024w_{2}^{4}-128w_{2}^{3}+656w_{2}^{2}$$

$$-320w_{2}+64,$$

$$L_{19,4}=768w_{1}^{2}w_{2}^{2}-192w_{1}^{2}w_{2}-1024w_{1}w_{2}^{4}-256w_{1}w_{2}^{3}+512w_{1}w_{2}^{2}-96w_{1}w_{2},$$

$$L_{19,5}=-1280w_{1}^{2}w_{2}^{2}+448w_{1}^{2}w_{2}-32w_{1}^{2}+1024w_{1}w_{2}^{4}+256w_{1}w_{2}^{3}-768w_{1}w_{2}^{2}$$

$$+416w_{1}w_{2}-64w_{1},$$

$$L_{19,6}=-1920w_{1}^{2}w_{2}^{2}+192w_{1}^{2}w_{2}+4096w_{1}w_{2}^{4}+1792w_{1}w_{2}^{3}-2048w_{1}w_{2}^{2}$$

$$+480w_{1}w_{2}-2048w_{2}^{6}-2048w_{2}^{5}+1536w_{2}^{4}+512w_{2}^{3}-736w_{2}^{2}+192w_{2}.$$
