# Supplementary material for: Fair curve designing by Said-Ball curve
Source: PLoS One. 2025 Jul 7;20(7):e0324553. doi: 10.1371/journal.pone.0324553 (PMC12233272; doi:10.1371/journal.pone.0324553)
Supplement: Attachment — Submitted filename: PONE D 23 25437 reviewer..pdf [file pone.0324553.s002.pdf]

The authors proposed a method for achieving fair curve using tangent continuous rational cubic Said-Ball curve.

The paper is well written with detail information in the appendix. However, the following points need to be addressed.

1. Under **Curvature variation energy,** I guess there is typo in the dimension of the matrix $L_{i,j}$. The matrix should be 19 by 6 not 15 by 6.
2. The authors said that the proposed method require less computation than previous methods [9, 15]. The justification of this statement could be more clearly if the authors can time taken to solve each example by the proposed method and that of the previous methods [9, 15].
3. In Table 6, example 8, the approximated curvature variation energy of the proposed method is greater than that of the method presented in [15]. Indicate that in the table or in the statement under Numerical results.
