# Supplementary material for: Fair curve designing by Said-Ball curve
Source: PLoS One. 2025 Jul 7;20(7):e0324553. doi: 10.1371/journal.pone.0324553 (PMC12233272; doi:10.1371/journal.pone.0324553)
Supplement: Attachment — Submitted filename: Response_to_Reviewers.docx [file pone.0324553.s004.docx]

**RESPONSE to REVIEWERS’**

**Paper Title:** Fair Curve Designing by Said-Ball Curve

**Authors:** Sana Zafar and Maria Hussain

**Manuscript ID:** PONE-D-23-25437

|  | **REVIEWER 1** | |
| --- | --- | --- |
| Sr.  No. | **Reviewer’s Comments** | **Author’s Response**  ***all added responses have been highlighted in yellow colour in the revised manuscript** |
| 1. | The authors can improve the paper with some examples and other related contents in this topic. | We the authors of the research paper are grateful to the reviewers for their valuable suggestions. Abstract has been improved to give the details of the research work. References [14, 15, 16, 20] have been added. The added references have been reviewed in introduction section 1. Examples for generation of 3D shapes have been added, see Fig. 9a, Fig. 9b and Fig. 10. |
| 2. | The abstract is poor, they can improve it. | As advised, the abstract has been revised and improved, see page 1, Abstract. |
| 3. | The introduction can be improved by adding more history. | New references [14, 15, 16, 20] have been added. The references have been reviewed in Section 1, page 1 and 2, paragraph 2(page1) and paragraph 1(page 2).  The significance of fair curves has been reinforced on Page 5, Section Curvature variation energy, paragraph 1. |
| 4. | The algorithms can be compared with others. | The proposed algorithms have been compared with [11] and [19] in Table 3 and 6. |
|  | **REVIEWER 2** | |
| 1. | The authors -proposed a method for achieving fair curve using tangent continuous rational cubic Said-Ball curve.  The paper is well written with detail information in the appendix. However, the following points need to be addressed.  Under **Curvature variation energy,** I guess there is typo in the dimension of the matrix $L_{i,j}$. The matrix should be 19 by 6 not 15 by 6. | It was a typing mistake in the matrix. It has been corrected. |
| 2. | The authors said that the proposed method requires less computation than previous methods [9, 15]. The justification of this statement could be more clearly if the authors can time taken to solve each example by the proposed method and that of the previous methods [9, 15]. | The proposed methods are algorithmically simple and time is calculated for two of the techniques presented in this research paper and is compared to the time elapsed in the references [11] and [19], see Table 3 and 6.  See page 8 and 10.  Since new references have been added which are highlighted in yellow in reference section so [9, 15] are now [11, 19]. |
| 3 | In Table 6, example 8, the approximated curvature variation energy of the proposed method is greater than that of the method presented in [15]. Indicate that in the table or in the statement under Numerical results. | We have indicated the energy for Example 8 below the Table 6 and explained the reason as well on Page 10, paragraph 1.  “Note that, in Table 6, Example 8 has the curvature variation energy greater than the previous method. The reason behind this greater value is the fact that the shape of the curve distorted for values lesser than 141 while interpolating with rational Said-Ball curve (1).” |
|  | **REVIEWER 3** | |
| 1 | The authors propose to fair rational Said-Ball's curves by means of strain energy and curvature variation energy.  Comments:  Define fair curve based on references: see Moreton's thesis: <https://www2.eecs.berkeley.edu/Pubs/TechRpts/1993/5219.html> | On Page 5, in section (ii) Curvature variation energy, the fair curve has been defined using the mentioned reference. It has been included in the reference list on number [20].  “Generally, a high degree of fairness is required. This concept of fairness is usually associated with the curvature of the underlying curve. A curve is considered fair if it consists of smoothly varying curvature having a few inflection points and curvature extrema. The curve that satisfies all these requirements is named as minimum variation curve MVC [20].” |
| 2 | Explain the motivation of choosing rational Said-Ball curve rather then NURBS or Beziers. | Motivation of using rational Said-Ball curve has been explained on page 2, section 2, paragraph 1  “Ball introduced a polynomial cubic curve which was slightly different from the ordinary cubic Bernstein basis. It was further used to design an aircraft design system [2]. The Ball functions are more efficient in computation than the Bernstein polynomials. Further it was proved that these possess the same shape preserving properties as the Bernstein basis. But the Said-Ball curve is better than Bézier curve for polynomial evaluation. Their representation better suited to degree raising and lowering than the Bézier representation [21]. The cubic Said-Ball curve uniquely approximates a function using its interpolation property but due to the absence of free parameters Said-Ball curve does not have the ability to modify the shape of the curve. Therefore, a rational function with two free parameters for the construction of fair curve. The introduced rational cubic Said-Ball curve(RCSBC) $P\left( t \right)$, is defined as follows:” |
| 3 | E_s : this is a well known arc length function, but you stated is as stretch energy However, eqn. (4) is squared which is the stretch energy, add refence. Its approximation is stated in Eqn.(5), show how you derived it. | We have added reference to equation (4) i.e. reference [8]. The reference [8] is a well-known published paper where equation (4) has been used.  Equation (5) is just first derivative of rational Said-Ball curve (1). |
| 4 | Eqn.(5); is it S^ tilde? In appendix, S^ tilde has u_i terms which are not defined. | Yes, it is $\tilde{S}.$ It is corrected on page 4 after (6).  The mathematical expressions for $\tilde{S_{i}}$ and $S_{i,j}$ are given in Appendix.  $u_{i}$ are defined on page 13 in detail. |
| 5 | Eqn.(7) has d_0 and d_1 with theta_0 and theta_1; define them. | These terms are now on **Page 4, paragraph 3**  “Here $d_{0}$ and$d_{1}$ are the norms of distances of control points $b_{0}, b_{1}$ and $b_{2}, b_{3}$ i.e. $d_{0}=\left\Vert b_{1}-b_{0} \right\Vert$, $d_{1}=\left\Vert b_{3}-b_{2} \right\Vert$. The $\theta_{0}$ and $\theta_{1}$ are the horizontal angles made be the curves at the initial and terminal points.” |
| 6 | Explain in brief what is 'fminunc' technique. | The MATLAB built-in function “fminunc” is has been explained Page 4 -5, last and first paragraphs.  “ The MATLAB built-in function … until the function converges to a minimum.” |
| 7 | Give reference to Eqn.(9 - 10). | Reference has been added on Page 5, it is [8]. |
| 8 | Eqn.(12); is it M^ tilde? In appendix, M^ tilde has u_i terms which are not defined. | These terms have been written in appendix now, see page 18. |
| 9 | The experiments shows the details of the resulted rational Said-Ball, however comparison to other types of curves are not carried out thus, we are unable to identify the effectiveness of the proposed method. | The proposed algorithms have been compared with [11] and [19] in Table 3 and 6.  Practical examples have been added, see Fig. 9a, 9b and 10. |
| 10 | Example of complex shape which involves piecewise curve is necessary to further concrete the proposed method. | We have constructed two examples of a glass and the character font “C” in Section 3.1.  The construction of these shapes has been explained on Page 10, in the last two paragraphs.  “In the end we present an application of the above proposed method in industrial design by constructing a glass. The surface of glass is constructed by using the control points and unit tangents at these points. The construction of the glass is hereby showing the efficiency. The profile curve of glass consists of four piecewise cubic curves. The surface of glass is generated by revolving this profile curve about z-axis.”  “An example of visually pleasing shape for the character “C” is established using the rational cubic Said-Ball curve. The character “C” is made up of ten segments. The control points and unit tangents are used to produce the shape of the character “C”. The corresponding final character of font “C” is given in Figure 10.”  The final shape of the two examples are obtained by joining the number of piecewise rational cubic Said-Ball curves. |
