# Supplementary material for: Fair curve designing by Said-Ball curve
Source: PLoS One. 2025 Jul 7;20(7):e0324553. doi: 10.1371/journal.pone.0324553 (PMC12233272; doi:10.1371/journal.pone.0324553)
Supplement: Attachment — Submitted filename: Response_to_Reviewers_auresp_2.docx [file pone.0324553.s005.docx]

**Authors’ Response to Reviewers**

**Paper Title:** Fair Curve Designing by Said-Ball Curve

**Reference No.:** PONE-D-23-25437R1

**Authors:** Sana Zafar and Maria Hussain

| **Sr.**  **No.** | **Reviewers’ Comments** | **Authors’ Response** |
| --- | --- | --- |
| 1. | The authors have corrected most of the parts. However, the following need further details for reproducibility: | The details have been provided for reproductivity. |
| 2. | Fig 9a: state clearly the control points of each segment and the continuity at the joint.  The proposed method involved stretch energy and the curvature variation energy which involves at least twice differentiable(G2), however segments are joined at G1, which is differentiable only once. Thus the combined piecewise become G1 continuous. | (i) Control points each segments have been provided in Table 7, Page 9.   Highlighted in yellow in the revised manuscript with track changes.   (ii) The proposed method assures $G^{1}$-continuiy, see page 2 & 3, section 2, equation (2) & (3).   The minimization of curvature variation energy avoids abrupt changes in curvature. The proposed numerical is not $G^{2}$-continuous. |
| 3. | Similarly, clearly show the details of the construction of Figure 10 and make sure it is G2 continuous.  Compare the metrics proposed in [11, 19] with your approach. | (i) The initial data and the computed value of the middle control points are given in Table 8 and 9. The detail of the construction of Figure 10 is provided on Page 9, Section 3 as follows:   “An example of visually pleasing shape for the character “C” is established using the rational cubic Said-Ball curve. Initial data for the construction of character “C” is given in the following Table 8.  The character “C” is made up of two segments, the segment above and below the x-axis. The control points and unit tangents are used to produce the shape of the character “C”. The computed values of control points $b_{1}$ and $b_{2}$ are given in Table 9. The corresponding final character of font “C” is given in Figure 10.”    Figure 10, is reconstructed  and a smooth character “C”  can be seen.   (ii) Comparison of the metrics of proposed method to the existing approaches [11, 19] is now provided in Table 3 and Table 6.   Example 1-4(stretch energy) are taken from [11).  Example 5-8(curvature variation energy) are taken from [19].  On page 9, Section 4, conclusions, the following details about the comparison is also added.  “Comparison of the metric of the proposed method(stretch energy and curvature variation energy) to the existing methods [11, 19] is given in Table 3 and Table 6. It shows that the methods proposed in this research paper have less stretch energy and curvature variation energy than the existing methods. Moreover, it works in fractions of seconds, so it is efficient as well.” |
| 4. | The application examples of piecewise curve and surface construction should be made at least G2 continuous, inline with the G2 fairing technique proposed. Currently the application example does not tally. | The proposed technique is $G^{1}$-continuous. Detail of the techniques is provided on page 2 and 3, section 2, Tangent Continuous Rational Cubic Said-Ball Curve.  The following is added on page 3, section 2 for explanation of the method.  “Substituting the values of control points from (2) and (3) in (1), a $G^{1}$-continuous rational cubic Said-Ball curve is obtained.” |
