# Supplementary material for: Fair curve designing by Said-Ball curve
Source: PLoS One. 2025 Jul 7;20(7):e0324553. doi: 10.1371/journal.pone.0324553 (PMC12233272; doi:10.1371/journal.pone.0324553)
Supplement: Attachment — Submitted filename: Response_to_Reviewers_auresp_3.docx [file pone.0324553.s006.docx]

**Authors’ Response to Reviewers**

**Paper Title:** Fair Curve Designing by Said-Ball Curve

**Authors:** Sana Zafar and Maria Hussain

| **Sr.**  **No.** | **Reviewers’ Comments** | **Authors’ Response** |
| --- | --- | --- |
|  | **Additional Editor Comments:**  The reviewer 4 raised some concerns on the innovation from a comparative perspective. Please systematically search relevant literature and add the comparison | It is tried to address all the concerns raised by the reviewer 4. Details are provided next. The relevant most recent literature has also been ad and comparison has been provided. Six new references has also been added. |
|  | **Reviewer # 3** | |
| 1. | The explanation of why the proposed method higher curvature variation energy for example 8 as compared to [19] unclear. Why the method fails to produce better RCSB for a simple shape? | The example 8 has been revised by improving optimization process. The improved value of curvature variation energy for example 8 is given Table 6 which is less than [19](now [21]). |
|  | **Reviewer # 4** | |
| 1. | The paper claims that the Said-Ball curve is more computationally efficient than the Bézier curve, but no evidence or benchmarking is provided. It remains unclear why this curve formulation is preferable over other rational Bézier-based curves. | The explanation has been provided on page 3, Section 2, paragraph 1 & 2  **Paragraph 1**  Ball introduced the cubic polynomials which reformed the manual lofting techniques of the CONSURF surface lofting programme [2]. It was used to design an aircraft design system [2]. The generalized Ball basis is normalized totally positive, and it possesses the shape-preserving property like Bernstein basis [9]. The recursive algorithm of Said-Ball curve for evaluating a polynomial curve runs twice as fast as the de Casteljau algorithm of Bézier curve, therefore the Said-Ball curve computationally efficient than the Bézier curve [23].  **Paragraph 2**  It is a special case of rational cubic Said-Ball curve [23] used to represent conics. |
| 2. | While the paper states that the method is computationally fast, it does not compare the computation time across different methods. | Computation time of both the proposed methods is provided in Table 3 & Table 6(page 8 & 9). The time is measured in seconds. It is clear from the table that the computation time of the proposed methods is fraction of seconds. |
| 3. | Why jerk energy used as a substitute for curvature variation? | The explanation is provided on page 5, last paragraph Formula of curvature of parametric curve $k\left( t \right)=\left( \frac{dx}{dt}\frac{d^{2}y}{dt^{2}}-\frac{dy}{dt}\frac{d^{2}x}{dt^{2}} \right)\left( \left( \frac{dx}{dt} \right)^{2}+\left( \frac{dy}{dt} \right)^{2} \right)^{-3/2}$involves radical terms, which has the computational complexity for evaluation of integral in (9). It also increases approximation error while evaluating the integral involved in (9) by numerical integration technique. Therefore, in literature the approximate form of the curvature variation energy is used which is known as jerk energy [10]. The jerk energy,$\hat{E}_{jerk}$, of the curve $P(t)$ is calculated as follows: \| $\hat{E}_{jerk}=\int_{0}^{1} \left\Vert P^{'''}\left( t \right) \right\Vert^{2} dt,$ \| (10) |
| 4. | The energy minimization functionals (stretch energy and curvature variation energy) lead to highly complex expressions. | The integration has been performed by programming Weddle’s rule through MATLAB software. It is a simple expression using results from numerical integration. Moreover, the unnecessary expression has been removed from the appendix. |
| 5. | The examples (glass and character “C”) are trivial and do not demonstrate any practical advantages in industrial applications. | The glass profile curve has continuously varying curvature, therefore it is used for validation of fair curve designing methods. Character “C” is a smooth curve and our method preserves it smoothness.  A new example of profile curve of ruddy duck in flight has been added. Data is taken from [5].  “In Figure 11 and 12, the top and bottom profile of a ruddy deck in flight is approximated respectively by the cubic Hermite interpolation method and the fair curve designing method proposed here. The data of the profile curve of the ruddy duck flight is taken from [5]. It is clear from Figure 11 that cubic Hermite fails to approximate the profile curve of the bird. But the fair curve designing method proposed in optimization problem-I approximated the profile curve approximately.” |
| 6. | The literature review is outdated and does not include recent advancements (post -2020) in fair curve design. | Authors are grateful to the reviewers for their comments. The following post-2020 references have added.  [6] Cantón, A., Fernández-Jambrina, L. and Vázquez-Gallo, M. J., Curvature of planar aesthetic curves, *Journal of Computational and Applied Mathematics*, **381**, (2021), 113042.  [9] Delgado, J. and Pe$\tilde{n}$a, J. M., On the generalized Ball bases, *Advances in Computational Mathematics*, **24**, (2006), 263-280.  [25] Said Mad Zain, S. A. A. A and Misro, M. Y., Shape analysis and fairness metric of generalized fractional Bézier curve, *Computational and Applied Mathematics*, **41(6)**, (2022), **276**.  [26] Saito, T. and Yoshida, N., Curvature monotonicity evaluation functions on rational Bézier curves, *Computers & Graphics*, **114**, (2023), 219-228.  [27] Wang, A., He, C., Song, Y. and Zhao, G., 2D Bézier curves with monotone curvature based on class A matrices, *Computer Aided Geometric Design*, **110**, (2024), 102279.  [29] Yang, X., Euler Bézier spirals and Euler B-spline spirals, *Computer Aided Geometric Design*, **112**, (2024), 102361.  After adding these references, the reference numbers have been changed. |
